# CD24–p53 axis suppresses diethylnitrosamine-induced hepatocellular carcinogenesis by sustaining intrahepatic macrophages

**DOI:** 10.1038/s41421-017-0007-9

**Published:** 2018-02-06

**Authors:** Dongling Li, Minling Hu, Ying Liu, Peiying Ye, Peishuang Du, Chi-Shan Li, Liang Cheng, Ping Liu, Jing Jiang, Lishan Su, Shengdian Wang, Pan Zheng, Yang Liu

**Affiliations:** 1 Key Laboratory of Infection and Immunity of CAS, Institute of Biophysics, Chinese Academy of Sciences, Beijing, China; 2 OncoImmune-Suzhou, Suzhou, China; 3 Center for Cancer and Immunology Research, Children’s Research Institute, Children’s National Health System and Department of Pediatrics, George Washington University School of Medicine, Washington, DC 20010 USA; 4 Institute of Biomedical Sciences, Academia Sinica, Taipei, Taiwan; 5 The first affiliated hospital, Jilin University, Changchun, China; 6 Lineberg Comprehensive Cancer Center, University of North Carolina, Chapel Hill, NC USA

## Abstract

It is generally assumed that inflammation following diethylnitrosamine (DEN) treatment promotes development of hepatocellular carcinoma (HCC) through the activity of intrahepatic macrophages. However, the tumor-promoting function of macrophages in the model has not been confirmed by either macrophage depletion or selective gene depletion in macrophages. Here we show that targeted mutation of *Cd24* dramatically increased HCC burden while reducing intrahepatic macrophages and DEN-induced hepatocyte apoptosis. Depletion of macrophages also increased HCC burden and reduced hepatocyte apoptosis, thus establishing macrophages as an innate effector recognizing DEN-induced damaged hepatocytes. Mechanistically, Cd24 deficiency increased the levels of p53 in macrophages, resulting in their depletion in *Cd24*^−/−^ mice following DEN treatment. These data demonstrate that the Cd24–p53 axis maintains intrahepatic macrophages, which can remove hepatocytes with DNA damage. Our data establish a critical role for macrophages in suppressing HCC development and call for an appraisal of the current dogma that intrahepatic macrophages promote HCC development.

## Introduction

Hepatocellular carcinoma (HCC) is the third most common cause of cancer-related deaths worldwide. Major risk factors for HCC include hepatitis B virus (HBV) and hepatitis C virus, aflatoxin B_1_, nonalcoholic steatohepatitis, chemical carcinogens (alcohol and nitrosamines) and genetic factors^1–3^. Accumulating epidemiological and clinical studies have provided convincing evidence that HCC is an inflammation-related cancer as >90% of HCCs are known to arise in the context of inflammation^4,5^.

Tumor-associated macrophages (TAMs) are a major component of the leukocytes infiltrating in tumors. The prevailing view is that TAMs have a critical role in promoting carcinogenesis and is based on several lines of clinical observations. First, increased TAM numbers correlate with angiogenesis, metastasis, and thus poor prognosis^6–9^. Consistent with this notion, activated monocytes/macrophages in the peritumoral stroma of HCC may promote angiogenesis and metastasis by expanding Th17 and IL-17-producing CD8^+^ T cells (Tc17 cells)^10–12^. Second, TAM numbers correlate with intratumoral regulatory T cells in HCC patients^13,14^. Third, increased expression of PD-L1 in Kupffer cells (KCs) or activated monocytes /macrophages is thought to contribute to the suppression of tumor-specific T-cell immunity in HCC tissues^15,16^. Fourth, monocytes/macrophages render natural killer cells dysfunctional in HCC through CD48/2B4 interaction^17^. Fifth, elevated serum levels of IL-6 have been frequently observed in patients with HCC^18^.

Corresponding with the clinical observations, data from several lines of preclinical investigations involving diethylnitrosamine (DEN)-induced HCC in mice are also consistent with an HCC-promoting function of inflammation and TAMs. For example, although deletion of the *IKKb* gene in hepatocytes markedly enhanced DEN-induced HCC, its deletion in both hepatocytes and leukocytes suppressed HCC development^19,20^. Although reduced NF-kβ activation in intrahepatic macrophages was proposed as the underlying mechanism for reduced HCC development, lineage-specific deletion was not technically possible to validate the hypothesis^19^. Likewise, IL-6, a major inflammatory cytokine, promoted tumorigenesis, and induced the expansion of cancer stem cells by activating the STAT3 signaling pathway^21,22^. *Il-6*^*−/*^^−^ mice or mice with selective IL-6 ablation in leukocytes exhibited a significant reduction of HCC development and eliminated the gender bias in liver cancer^23,24^. Again, although the genetic defect was attributed to intrahepatic macrophages, such selective deletion cannot be achieved based on the experimental design. Therefore, the putative contribution of intrahepatic macrophages to HCC carcinogenesis deserves further investigation.

Paradoxically, targeted mutation of key regulators of inflammatory responses, such as the *Tlr2* and *Tlr4* genes, which reduces intrahepatic inflammatory responses, has been shown to dramatically promote DEN-induced HCC^25,26^, although several groups reported that germline deletion of either *Tlr4* or *Tlr2* decreased HCC development^27,28^. The *Tlr4* mutation appears to affect DNA damage and repair as the expression of Ku70 was also reduced^25^. DNA damage occurs at an early stage of carcinogenesis in normal parenchyma liver cells^29^. While the cell-intrinsic mechanisms could repair DNA damage or eliminate cells with unrepaired damage^30^, the innate immune system may also eliminate cells with DNA damage^31^ or alter DNA repair through unspecified mechanisms^25^. These data raised the intriguing possibility that the innate immune system may protect against DEN-induced HCC carcinogenesis.

CD24 is a glycosylphosphatidylinositol (GPI)-anchored cell surface protein with a broad expression in hematopoietic cells and cancer cells^32^. *Cd24* acts as a costimulatory molecule to promote adaptive immunity including autoimmunity^33–37^, but represses innate immune response to liver injuries caused by acetaminophen^38^. Given the role for *Cd24* in regulating of inflammation to liver injuries^38^, we investigated the impact of *Cd24* deletion on HCC carcinogenesis. Surprisingly, targeted mutation of *Cd24* reduced the number of F4/80^+^ cells and reduced F4/80^+^ cell-mediated apoptosis of hepatocytes, resulting in markedly increased host susceptibility to DEN-induced HCC. Consistent with a protective function of macrophages against HCC carcinogenesis, we show depletion of macrophages at DEN induction, or 5 months after induction, increased the incidence and tumor size of HCC. Our data provide a new model on the evolving function of macrophages during HCC pathogenesis and call for the prevailing view of macrophage function in DEN-induced HCC to be re-evaluated.

## Results

### Targeted mutation of *Cd24* promotes hepatocarcinogenesis

**Fig. 1:**
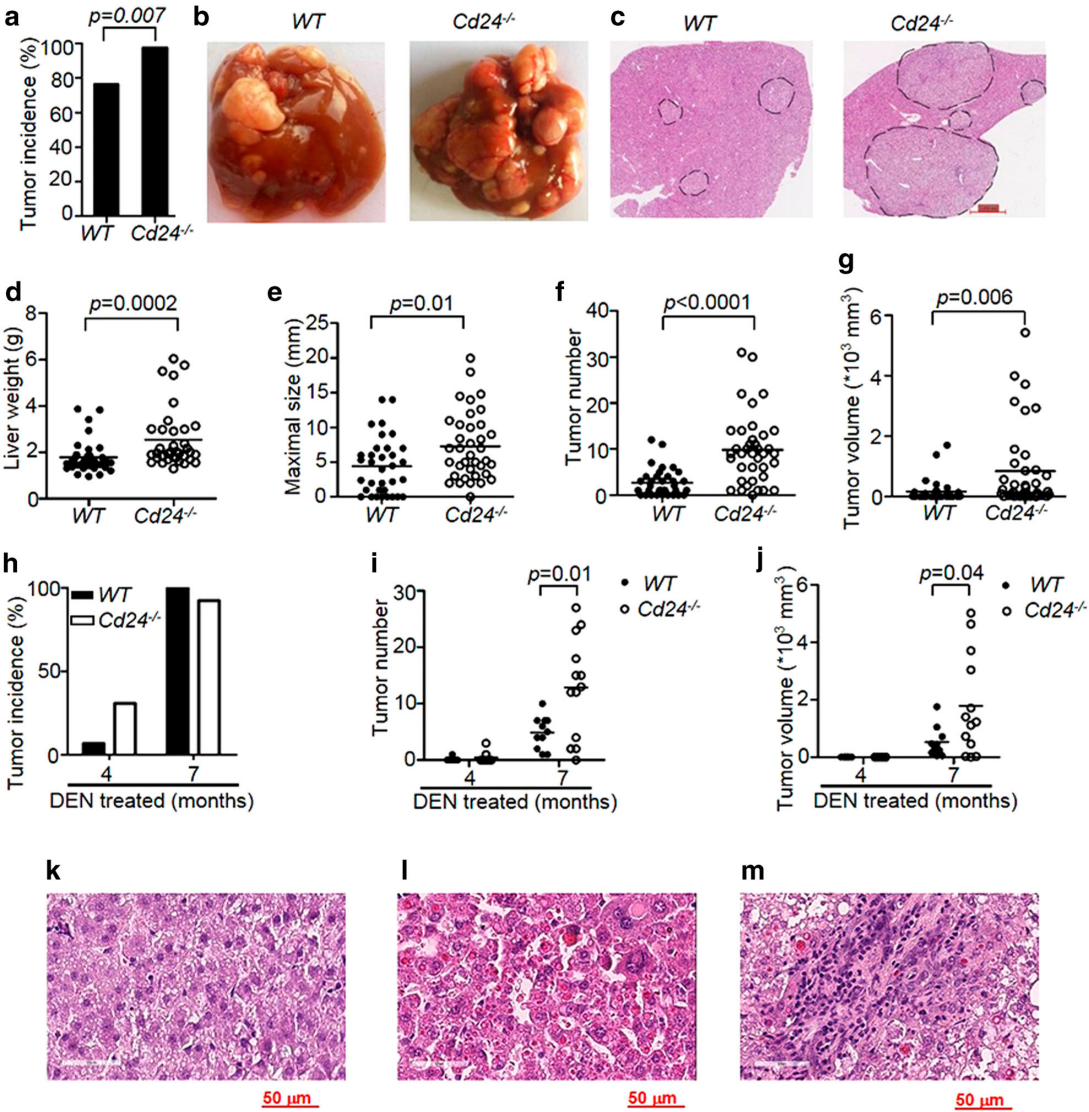
Targeted mutation of *Cd24* promotes the development of HCC carcinogenesis. **a** Incidence of HCC tumors (diameter ≥1.0 mm) in male WT (*n* = 34) and *Cd24*^−/−^ (*n* = 38) mice 8 months after 15 μg/g DEN administration to 15-day-old mice. **b** Representative gross liver anatomy of 8-month-old DEN-treated male WT (left) and *Cd24*^−/−^ mice (right). **c** Haematoxylin and eosin (**h**&**e**) staining of liver sections (scale bar = 1 mm) in 8-month-old DEN-treated male WT (left) and *Cd24*^−/−^ mice (right). Black dash lines indicate border of the tumor nodules. **d**–**g** Liver weight **d**, maximum tumor diameters **e**, numbers of ≥1.0 mm visible tumors per mouse **f**, and total tumor volumes **g** in male mice 8 months after DEN (15 µg/g) injection. Data are pooled from multiple experiments. **h**–**j** Incidence of mice with liver tumors **h**, tumor numbers **i**, total tumor volumes **j** in male mice at the 4th or 7th month after DEN (15 µg/g) injection. Two pooled independent experiments. Data represent means. *p < 0.05, **p < 0.01, ***p < 0.001, using two-tailed, unpaired Student’s *t-*test **e**–**g**, **h**–**j** or Mann–Whitney test **d**. **k**–**m** High power (× 40) H&E image of tumor nodules in livers from WT **k** and *Cd24*^−/−^ **l**, **m** mice. Note benign morphology of WT adenoma cells **k**, pleomorphic nuclei and trabecular carcinoma **l** and leukocyte infiltration **m** into *Cd24*^−/−^ HCC. Data shown are sections prepared at 8 months after DEN treatment

To investigate the role for *Cd24* in DEN-induced carcinogenesis, *Cd24*^*−/−*^ mice and their WT littermates were injected with 15 μg/g DEN on day 15 after birth. Previous studies revealed no spontaneous liver dysfunction or HCC in *Cd24*^−*/−*^ mice for up to 2 years^39^. Within 8 months of DEN treatment, almost all the *Cd24*^*−/−*^ male mice (37/38) developed typical HCC, whereas only 76.5% of WT male mice (26/34) bore tumors (Fig. 1a). Gross anatomy (Fig. 1b) and Haematoxylin and eosin (H&E) analysis (Fig. 1c) revealed that targeted mutation of *Cd24* drastically increased the number and the size of HCC lesions. At 8 months, liver weights were significantly increased in *Cd24*^−^^*/*^^−^ mice (2.5 ± 0.2 g) compared with those in WT mice (1.8 ± 0.1 g) (*p* = 0.0002) (Fig. 1d). The increase in liver weight is attributable to increased tumor size, as the diameters of the largest tumors in *Cd24*^−/−^ mice (7.7 ± 0.9) were nearly twice as those in the WT littermates (4.4 ± 0.7) (*p* = 0.0057) (Fig. 1e). Moreover, the numbers of observable HCC lesions were significantly higher in *Cd24*^−/−^ mice (9.8 ± 1.2) than in WT mice (2.7 ± 0.5) (*p *< 0.0001) (Fig. 1f). When all tumors from one mouse were combined, *Cd24*^−/−^ mice bore a 5.1-fold larger tumor volume than WT mice (844.8 ± 219.3 *vs* 166.4 ± 64.4, *p* = 0.0002) (Fig. 1g). To further evaluate the impact of the *Cd24* deletion on HCC onset and progression, we killed mice at 4 and 7 months. As shown in Fig. 1h, at 4 months, only 1/15 WT mice developed HCC, whereas 4/13 *Cd24*^−/−^ mice developed visible HCC. While essentially all *Cd24*^−/−^ mice had visible tumors at 7 months in this experiment, *Cd24*^−/−^ livers had more tumors than WT livers, as measured either by the numbers of observable HCC lesions (Fig. 1i) or by the total tumor volumes (Fig. 1j).

High-power histology analyses revealed that in addition to the differences in tumor sizes and incidences, *Cd24* deficiency increased malignancy of liver tumors (Fig. 1k–m). At 8 months after DEN treatment, tumor cells found in WT mice were benign as the nuclei/cytoplasm ratios are small (Fig. 1k). In contrast, the tumor cells in the *Cd24*-deficient mice exhibit large and pleomorphic nuclei (Fig. 1l). Moreover, trabecular carcinoma (Fig. 1l) and lesions with lymphocyte infiltration (Fig. 1m) were also found in some tumors from the *Cd24*^−/−^ mice. Apart from alteration in cellular morphology, we also observed extensive architecture changes in tumors from *Cd24*^−/−^ but not WT mice (Supplementary Figure S1). These include ductules extending to the peripheries of foci lesions and associated with bile ducts (Supplementary Figure S1a, black arrow), foci of lesions penetrated into the walls and proliferated in the lumens of a hepatic vein branch (Supplementary Figure S1b, red arrow), and vascularization by portal vein branches (Supplementary Figure S1c, blue arrow). These features further support the idea that *Cd24* deficiency promotes HCC development. The striking impact of germline deletion of the *Cd24* gene in HCC onset and progression suggests that *Cd24* is a critical repressor for carcinogenesis of HCC.

### Reduction in acute inflammation and hepatocyte apoptosis in *Cd24*^−/−^ mice

**Fig. 2:**
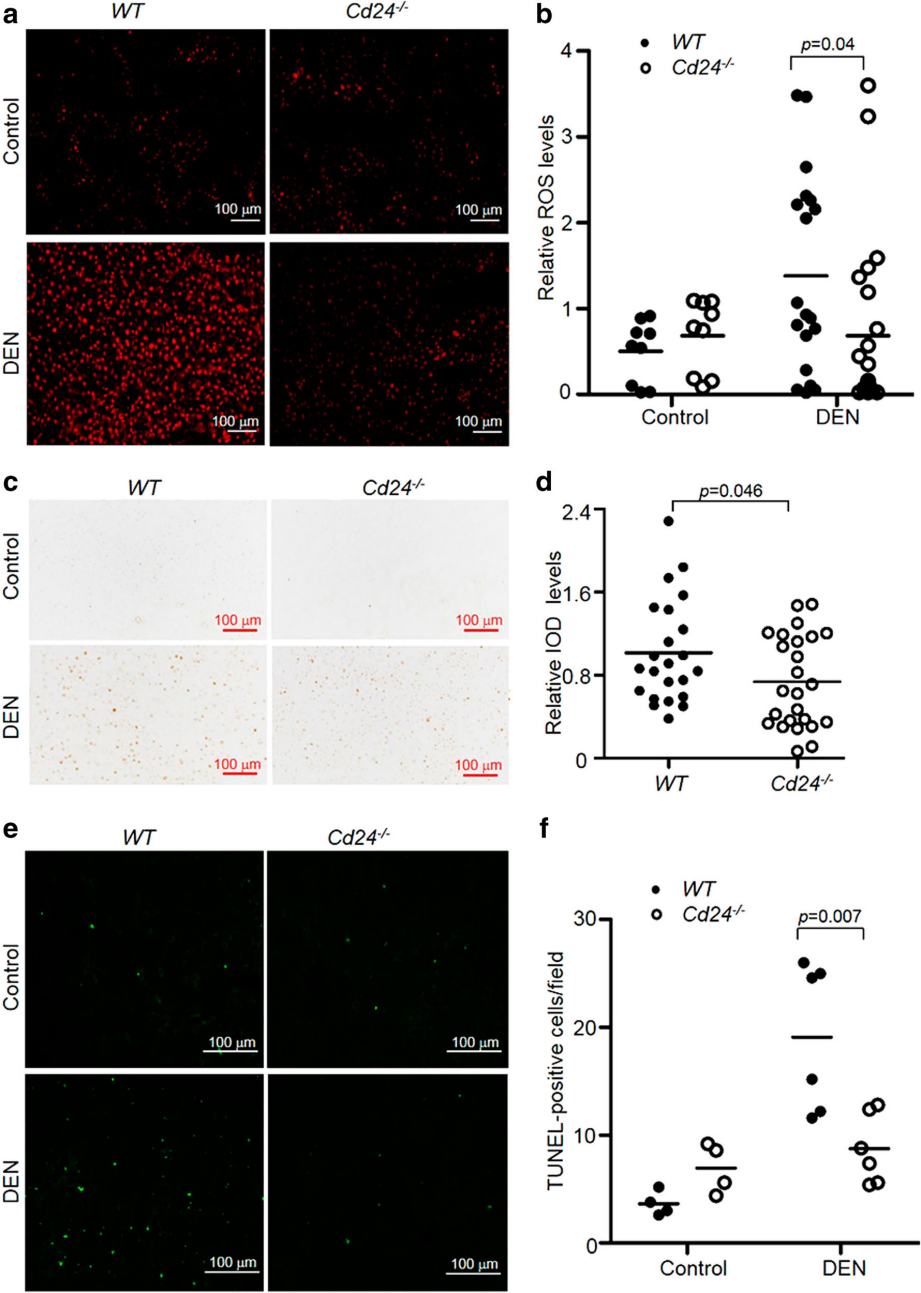
*Cd24* deficiency reduces ROS production and apoptosis of hepatocytes. Liver tissues were isolated at 24 h after 15 μg/g DEN injection into 15-day-old male mice for histological staining. **a** and **b** Liver cryosections were incubated with 2 μM dihydroethidine for 30 min at 37 °C. Cells staining positive (red) for the oxidized dye were identified by fluorescence microscopy **a** and quantified by Image-Pro Plus 6.0 software **b**. At least 10 fields from different mice were counted. Data from two independent experiments are pooled (Control mice: *n* = 9; DEN-mice: *n* = 19 or 23). **c** γ-H2A.X staining for DNA damage in DEN-treated mice. Liver sections were obtained at 24 h after DEN treatment. **d** Relative Integrated optical density (IOD) of γ-H2A.X-positive cells quantified by Image-Pro Plus 6.0 software. At least three slides from successive sections of different mice were analyzed. Data from two independent experiments are pooled (*n* = 23 or 25). **e** Representative liver sections stained by the TUNEL assay. **f** Average numbers of TUNEL-positive cells in a high-power field of a fluroscerence microscope. At least five fields from different mice were counted. Data represent the representative experiments from three independent experiments (Control mice: *n* = 4; DEN-mice: *n* = 6).. Scale bar = 100 μm. The data were analyzed by Student’s *t*-test. Data are means

Reactive oxygen species (ROS) are the major effector molecules produced by neutrophils and activated macrophages^40,41^. As the first test to evaluate the effect of acute inflammation on HCC, we evaluated the levels of ROS in WT and *Cd24*^−/−^ liver sections. As shown in Fig. 2a and quantified in Fig. 2b, whereas comparable levels of ROS staining were found in WT and *Cd24*^−/−^ livers from control mice that received no DEN, *Cd24*^−/−^ sections showed much reduced ROS signals 24 h after DEN administration. Interestingly, although ROS^+^ cells in the untreated groups and those isolated from 1 and 4 months after DEN treatment had the morphology of hepatocytes (Supplementary Figure S2), immediately after DEN treatment they became mostly mononuclear cells based on the cellular size. Therefore, reduction in ROS levels in the *Cd24*^−/−^ mice may reflect a reduction in inflammation. In addition, we also evaluated ROS-induced DNA damage based on the analysis of γ-H2AX expression in liver tissues. Our data demonstrated that *Cd24*^−/−^ mouse livers showed a significant decrease in γ-H2AX levels when compared with WT mouse livers (Fig. 2c, d).

As ROS is a major mediator for the induction of apoptosis, we evaluated apoptosis in liver sections using TUNEL staining. As shown in Fig. 2e, f, DEN-treated WT liver sections contained a high number of apoptotic hepatocytes on day 1 after DEN treatment. This number is much lower in the *Cd24*^−/−^ liver sections. To evaluate if ROS is responsible for the death of hepatocytes, we pre-treated mice with hydroxyanisole (BHA) to quench ROS and then measured the number of TUNEL^+^ cells at 24 h after DEN treatment. As shown in Supplementary Figure S3, BHA reduced the percentage of apoptotic cells in the WT but not *Cd24*^−/−^ cells. Therefore, the elevated ROS in the WT mice contributed to increased apoptosis of liver cells one day after DEN administration.

Recent studies have shown that depletion of adaptive immune cells promotes formation and progression of DEN-induced liver cancer^42^. In order to determine the potential role of immune cells in HCC initiation, we isolated liver mononuclear cells and analyzed the phenotype of inflammatory cells in the livers of *Cd24*^−/−^ and WT mice by FACS analysis. We found that CD11b^+^ (Supplementary Figure S4A and B) were increased in WT but not in *Cd24*^−/−^ mice after DEN treatment. In contrast, the percentage of T cells, including both CD4 and CD8 T cells, was significantly higher in *Cd24*^−/−^ livers than in that of WT mice, with or without DEN treatment (Supplementary Figure S4C and D). Higher levels of adaptive responders may also contribute to increased carcinogenesis.

**Fig. 3:**
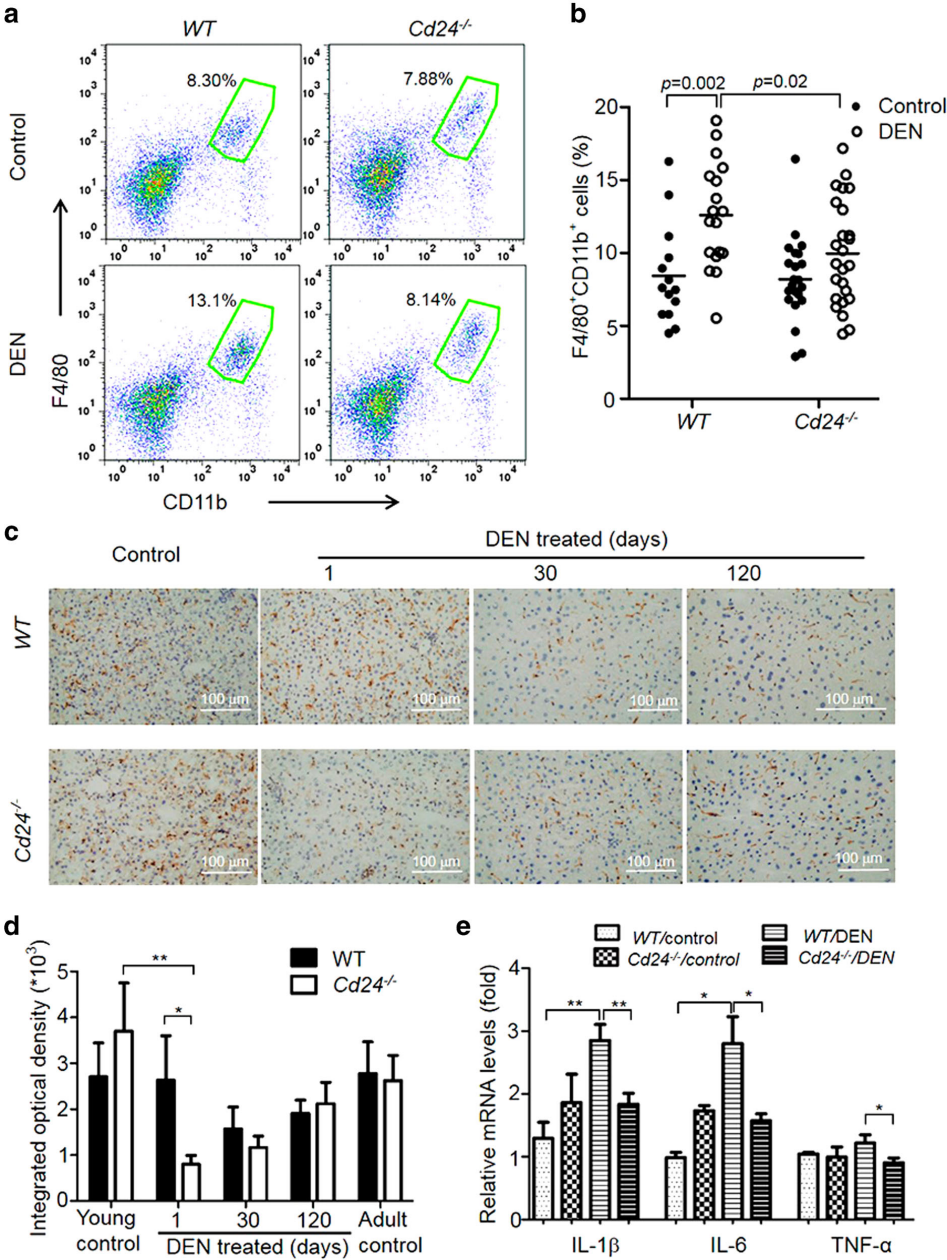
*Cd24* deficiency reduces acute inflammation caused by DEN treatment. **a**, **b** Flow cytometry analysis (FACS) of intrahepatic F4/80^+^ cells. Liver mononuclear cells were isolated 24 h after 15 μg/g DEN or 0.9% NaCl solution (as control) by intraperitoneal injection to 15-day-old mice for FACS. **a** The data are representative FACS profiles. **b** Percentages of F4/80^+^CD11b^+^ cells among CD45^+^ cells in liver. Data are pooled from multiple experiments involving age and gender matching mice. **c** and **d** *Cd24* deficiency decreases the number of intrahepatic F4/80^+^ cells. Liver tissues were isolated at the indicated timepoints after 15 μg/g DEN injection into 15-day-old mice for the F4/80 staining. The data in **c** are representative images of F4/80 staining (scale bar = 100 µm), and those in **d** are the mean ± SEM. Integrated optical density of F4/80-positive cells quantified by Image-Pro Plus 6.0 software in a low-power field of a microscopy. At least five fields each section were counted. Data are from one of two independent experiments (Control: *n* = 5; DEN-mice: *n* = 9). **e** The inflammatory cytokine mRNA transcript levels were determined by Real-time PCR. Liver tissues were isolated 24 h after 15 μg/g DEN injection into 15-day-old mice for cytokine measurement. Control: *n* = 5; DEN-mice: *n* = 11. Data are means ± SEM, and are pooled from independent experiments

Importantly, as shown in Fig. 3a, b, although the percentage of macrophages/KCs (F4/80^+^CD11b^+^) was increased in DEN-treated WT livers, DEN treatment failed to increase the frequency of macrophages in *Cd24*^−/−^ livers. A more marked difference was observed by immunohistochemistry (IHC) analysis, which revealed nearly threefold reduction in F4/80^+^ cells from *Cd24*^−/−^ livers on day 1 after DEN treatment compared with WT livers (Fig. 3c, d). The reduction is transient as it is not observed on day 30 and beyond when F4/80 + cell numbers in WT liver return to lower levels (Fig. 3c, d). Based on markers used in previous studies^43^, we further identified, by FACS analysis, the percentage of liver-resident KCs (defined as F4/80^+^CD11b^lo^Ly6C^lo^). We found that it was increased following DEN treatment in WT livers but not in *Cd24*^−/−^ livers (Supplementary Figure S5A and B). In contrast, the percentage of monocyte-derived macrophages (MoMs, defined as F4/80^+^CD11b^hi^Ly6C^int^) was not obviously changed in either WT or *Cd24*^−/−^ livers following DEN treatment (Supplementary Figure S5A and C). These data revealed selective decrease of the intrahepatic KCs in the *Cd24*^−/−^ mice shortly after DEN treatment. However, whereas both flow cytometry result of isolated mononuclear cells and IHC analysis of liver sections revealed the impact of *Cd24* deletion on acute inflammatory cells, only IHC reveals a net reduction of intrahepatic macrophages in *Cd24*^−/−^ livers after DEN treatment. This is perhaps owing to an uneven loss of F4/80^+^ cells during FACS isolation of mononuclear cells in different groups. Despite of the subtle differences, both methods reveal a critical role for *Cd24* in the maintenance of high numbers of intrahepatic F4/80^+^ cells in DEN-treated mice at the time of DEN induction.

To substantiate the contribution of *Cd24* to acute inflammation, we measured the amounts of *Il-6*, *Il1b*, *Tnfa*, and *Mcp-1* transcripts in untreated and DEN-treated mice. As shown in Fig. 3e, whereas elevation of *Il-6*, *Il1b,* and *Tnfa* transcripts was observed following DEN treatment in WT livers, no increase was found in the *Cd24*^−/−^ livers. Reduction of IL-1β protein was also observed in the *Cd24*^−/−^ livers upon DEN treatment (Supplementary Figure S6A). However, *Cd24* deletion had no effect on the expression of *Mcp-1* after DEN administration (Supplementary Figure S6B). Corresponding to a reduced inflammatory response, STAT3 phosphorylation was reduced in *Cd24*^−/−^ liver tissues after DEN administration (Supplementary Figure S6C). Furthermore, although WT mice exhibited a significant increase in serum HMGB1 levels at 24 h after DEN treatment (Supplementary Figure S6D), no increase was found in the sera from *Cd24*^−/−^ mice. HMGB1 can be secreted actively by activated leukocytes and released passively by necrotic cells. As there were no significant changes in serum ALT levels, a marker of acute liver toxicity after DEN injection in WT and *Cd24*^−/−^ mice (Supplementary Figure S6E), it is more likely that the increased HMGB1 may be secreted from activated leukocytes, but not necrotic hepatocytes.

We next addressed the effects of *Cd24* deficiency in chronic response. We detected a persistent increase in ROS accumulation in livers of *Cd24*^−/−^ mice compared with WT livers 1 and 4 months after DEN administration (Supplementary Figure S2A and B). However, compared with WT mice after DEN treatment, *Cd24*^−/−^ mice displayed a persistent decrease in cell apoptosis in liver as marked by TUNEL staining (Supplementary Figure S7A and B). In contrast, *Cd24*^−/−^ livers showed a remarkable increase in proliferation biomarker PCNA (proliferating cell nuclear antigen) as indicated by IHC staining (Supplementary Figure S7C and D). Except for IL-6, no significant differences were observed between WT and *Cd24*^−/−^ mice for inflammatory cytokines (Supplementary Figure S7E). These results suggest that *Cd24* deficiency may promote HCC by protecting liver cells from DEN-induced apoptosis and increasing cell proliferation.

### Intrahepatic macrophages induce apoptosis of hepatocytes and suppress hepatocellular adenoma in DEN-treated mice

**Fig. 4:**
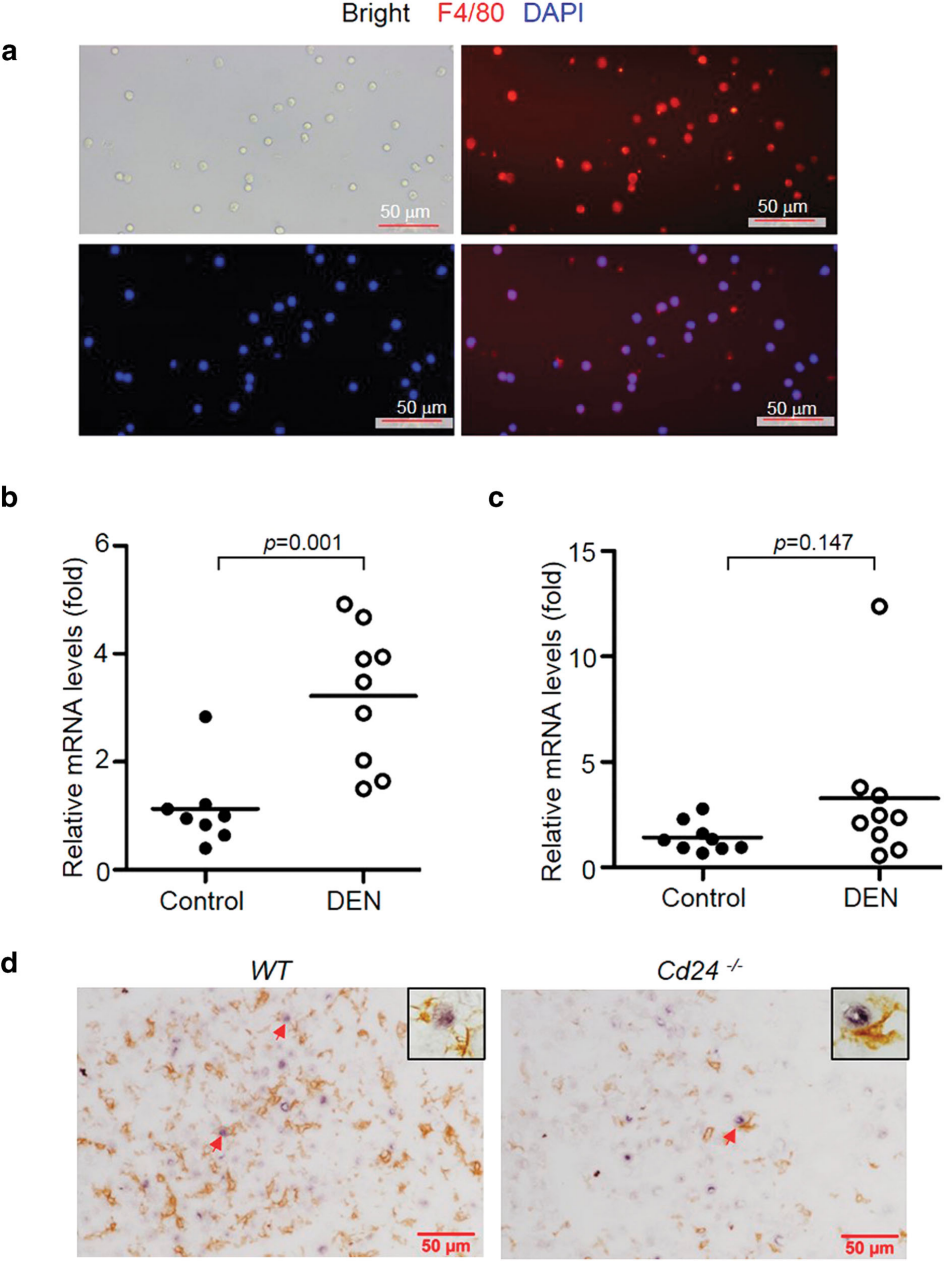
Cd24 deficiency ablates acute iNOS accumulation in intrahepatic macrophages after DEN treatment. **a** Purity characterization of isolated intrahepatic macrophages. The isolated liver mononuclear cells were incubated for 2 h to remove the non-adherent cells. The adherent cells were used as source for intrahepatic macrophages that were stained with anti-F4/80 antibodies by immunocytochemical method. **b**, **c** Quantitative RT-PCR of iNOS expression in isolated live macrophages/Kupffer cells from mice 24 h after 15 μg/g DEN injection at 15 days of age. **b** WT mice. **c** *Cd24*^−/−^ mice. The data were analyzed by Student’s *t*-test. Data from individual mice in two independent experiments are presented. **d** Liver macrophages/Kupffer cells reside in close proximity to DNA-damaged cells identified by γ-H2AX. Representive photograph of F4/80 (brown) and γ-H2AX (blue) double staining in frozen liver sections from mice 24 h after 15 μg/g DEN injection at 15 days of age. Red arrows indicate co-staining for F4/80 and γ-H2AX

To determine whether DEN treatment induces iNOS expression in macrophages, we isolated liver macrophages/KCs from DEN-treated mice. The purity of macrophages after isolation was >90% based on immunofluorescence microscopy result (Fig. 4a). A significant increase in iNOS mRNA levels was observed in DEN-treated WT intrahepatic macrophages (Fig. 4b). In contrast, no increase was found in the livers of DEN-treated *Cd24*^−/−^ mice (Fig. 4c). Furthermore, we also observed that liver macrophages/KCs resided in close proximity to DNA-damaged cells identified by γ-H2AX (Fig. 4d).

**Fig. 5:**
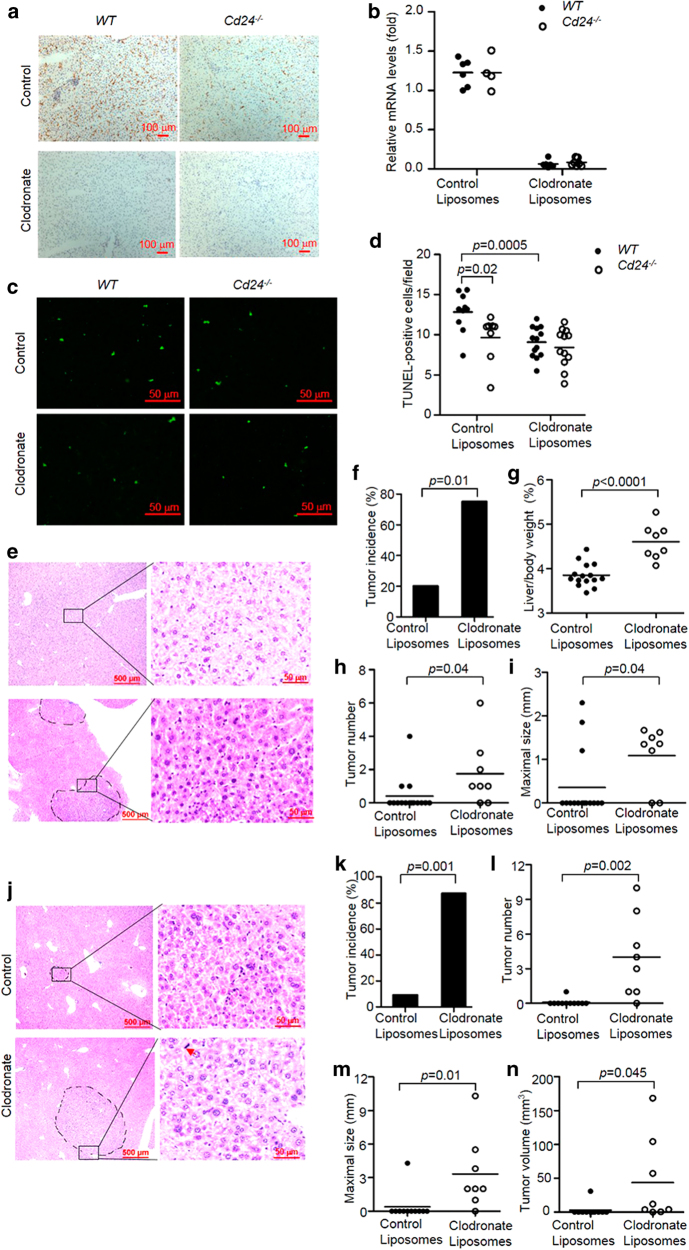
Intrahepatic macrophages are responsible for increased apoptosis of WT but not *Cd24*^−/−^ mouse livers and for suppression of HCC development. To deplete liver macrophages, 13-day-old mice were injected i.p. with 70 μL of liposomal clodronate or control at 48 h before 15 μg/g DEN administration. **a** F4/80 staining for liver macrophages following injection of control liposomes and clodronate liposomes into WT and *Cd24*^−/−^ mice. Scale bar = 100 μm. **b** mRNA levels of F4/80-encoding *Emr1* were analyzed by RT-PCR. **c** Liver sections were analyzed by TUNEL assay. Scale bar = 100 μm. **d** Average numbers of TUNEL-positive cells in a high-power field of a fluroscerent microscope. At least 10 fields from different section were counted. Control liposomes: *n* = 8; Clodronate liposomes: *n* = 12. Representative data from one of two experiments are shown. The data were analyzed by Student’s *t*-test. (**e**–**i** Depletion of macrophage at the time of DEN induction increases the incidence of HCC. Liver macrophages in 13-day-old WT mice were depleted by injection i.p. with 70 µl of clodronate or control liposomes twice within 5 days. 48 h after the first injection of liposomes, mice were treated with 15 µg/g DEN. Mice in both groups were kept for 5 additional months, after which they were killed and analyzed to determine tumor load and morphology. (**e** Histology analyses revealed significant difference in neoplastic transformation of adenomas from control liposome-treated (top panels) or clodronate-treated macrophage-depleted mice (bottom panels). The left panels show 10 × images, whereas the right panels show high-power images. Note enlarged and pleomorphic nuclei in the adenoma from the macrophage-depleted mice. **f** Tumor incidences (diameter ≥1.0 mm), **g** liver to body weight ratios, **h** tumor numbers, and **i** maximal tumor sizes are shown. Control liposomes: *n* = 15; Clodronate liposomes: *n* = 8. Data from two pooled independent experiments are shown. **j**–**n** WT mice were injected with 15 μg/g DEN at 15-day of ages. After 5 months, 200 μL of a 1:1 PBS-diluted liposomes were injected via the tail vein once every 5 days for a total of three times. All mice were killed 1 month after the last injection. (**j**) Histology analyses revealed significant difference in neoplastic transformation of adenomas from control liposome-treated (top panels) or clodronate-treated macrophage-depleted mice (bottom panels). The left panels show 10 × images, whereas the right panels show high-power images. Note: more heterogenous, enlarged, and pleomorphic nuclei as well as mitosis (red arrow) in adenoma from the macrophage-depleted but not control liposome-treated mice. (**k**) Tumor incidences (diameter ≥1.0 mm), **l** tumor numbers, **m** maximal tumor sizes, and **n** total tumor volumes were shown. Control liposomes: *n* = 11; Clodronate liposomes: *n* = 8. The data were analyzed by Student’s *t*-test

To further confirm that liver macrophages/KCs are responsible for the hepatocyte apoptosis in DEN-treated livers, WT and *Cd24*^−/−^ mice were treated with control liposomes or liposomal clodronate to deplete macrophages/KCs starting at 48 h before DEN injection. KCs are depleted as early as 12 h after administration with liposome-encapsulated clodronates, and repopulation starts at the end of the first week^44^. As shown in Fig. 5a, there was almost complete depletion of liver macrophages in both WT and *Cd24*^−/−^ mice. The depletion was also confirmed by F4/80-encoding *Emr1* mRNA levels (Fig. 5b). Consistent with the published data^44^, depletion of KCs suppressed the induced mRNA levels of *Il1b* and *Il6* and slightly increased the levels of *Mcp-1* (Supplementary Figure S8). TUNEL assay showed that liver macrophage depletion resulted in diminished apoptosis in DEN-treated WT livers, but had no effect on apoptosis in *Cd24*^−/−^ livers. Importantly, no significant differences existed in the number of apoptotic cells between WT and *Cd24*^−/−^ livers after liver macrophages were depleted (Fig. 5c, d). These data demonstrate that intrahepatic macrophages are responsible for the hepatocyte apoptosis in DEN-treated WT mice and that the reduction in macrophages in the *Cd24*^−/−^ livers is responsible for the decreased hepatocyte apoptosis following DEN treatment.

To examine whether intrahepatic macrophage accumulation in DEN-treated WT mice is involved in the inhibition of tumor incidence and growth, clodronate-containing or control liposomes were administered to WT mice to deplete intrahepatic macrophages 48 h before DEN injection at 13 days of age. Mice in both groups were kept for five additional months, after which they were killed and analyzed to determine tumor burden. As expected, at this early time point, no architectural alteration was observed in tumors from either WT or *Cd24*^−/−^, and the tumors were mostly hepatocellular adenoma (Fig. 5e). However, the tumor cells in *Cd24*^−/−^ adenoma were considerably more advanced in malignant transformation based on the nuclei/plasma ratio and by the pleomorphic nuclei and the presence of mitotic tumor cells (Fig. 5e). Summary data were presented in Fig. 5f–i. As shown in Fig. 5f, clodronate administration markedly increased the incidence of macroscopic liver adenoma from 20% (3/15) to 75% (6/8). Furthermore, the ratio of liver to body weight (Fig. 5g), number of tumors (Fig. 5h), and maximum tumor sizes (Fig. 5i) were significantly larger in clodronate-administrated WT mice than in the control treated mice. These data indicate that intrahepatic macrophage depletion at DEN induction promotes incidence and growth of HCC.

Accumulating evidence indicates that macrophage counts are significantly higher in peritumoral liver tissues than in the corresponding intratumoral tissues^45,46^. By immunohistochemical staining using an anti-F4/80 antibody, we confirmed the enhanced accumulation of macrophages in peritumoral liver tissues during DEN-induced hepatocarcinogenesis compared with intratumoral liver tissues (Supplementary Figure S9). To further investigate the role of intrahepatic macrophages after tumor formation, we depleted the intrahepatic macrophages using liposomal clodronate 5 months after DEN treatment when HCC was visible in some mice and killed the mice one month later for analysis. The data were presented in Fig. 5j–n. Histologically, the cells in the tumor nodules in control liposome-treated mice lacked features of neoplastic transformation, as the nuclei were not significantly enlarged and relatively uniform. In contrast, significant heterogeneity was observed in the sizes of nuclei in adenoma in mice with macrophage depletion, and many of the nuclei were pleomorphic (Fig. 5j), suggesting that depletion of macrophages at 5 months could still accelerate malignant transformation of the hepatocellular adenoma. Strikingly, the incidence of macroscopic liver cancers in clodronate-treated mice was 87.5% (7/8), whereas that in the control liposome group was only 9.1% (1/11) (Fig. 5k). The number of tumors (Fig. 5l), maximal tumor sizes (Fig. 5m), and total tumor volumes (Fig. 5n) in clodronate-administrated mice were also much increased when compared with control mice. These data suggested that TAM also inhibit HCC progression.

Together, these data indicate that intrahepatic macrophage depletion at either at DEN induction or 5 months after DEN induction promotes incidence and growth of HCC, although the functions of macrophages at these different timepoints were not necessarily the same. As chronic treatment with clodronate could affect hepatocyte function, it is not possible to continue the treatment until the mice developed HCC. Inshort, intrahepatic macrophages have an important protective role against DEN-induced HCC. It remains to be investigated whether the protective functions of macrophages against HCC carcinogenesis are achieved through different mechanisms to that in tumor-bearing mice at different times after DEN treatment.

### DEN treatment induces depletion of *Cd24*^−/−^ macrophages by a p53-dependent mechanism

**Fig. 6:**
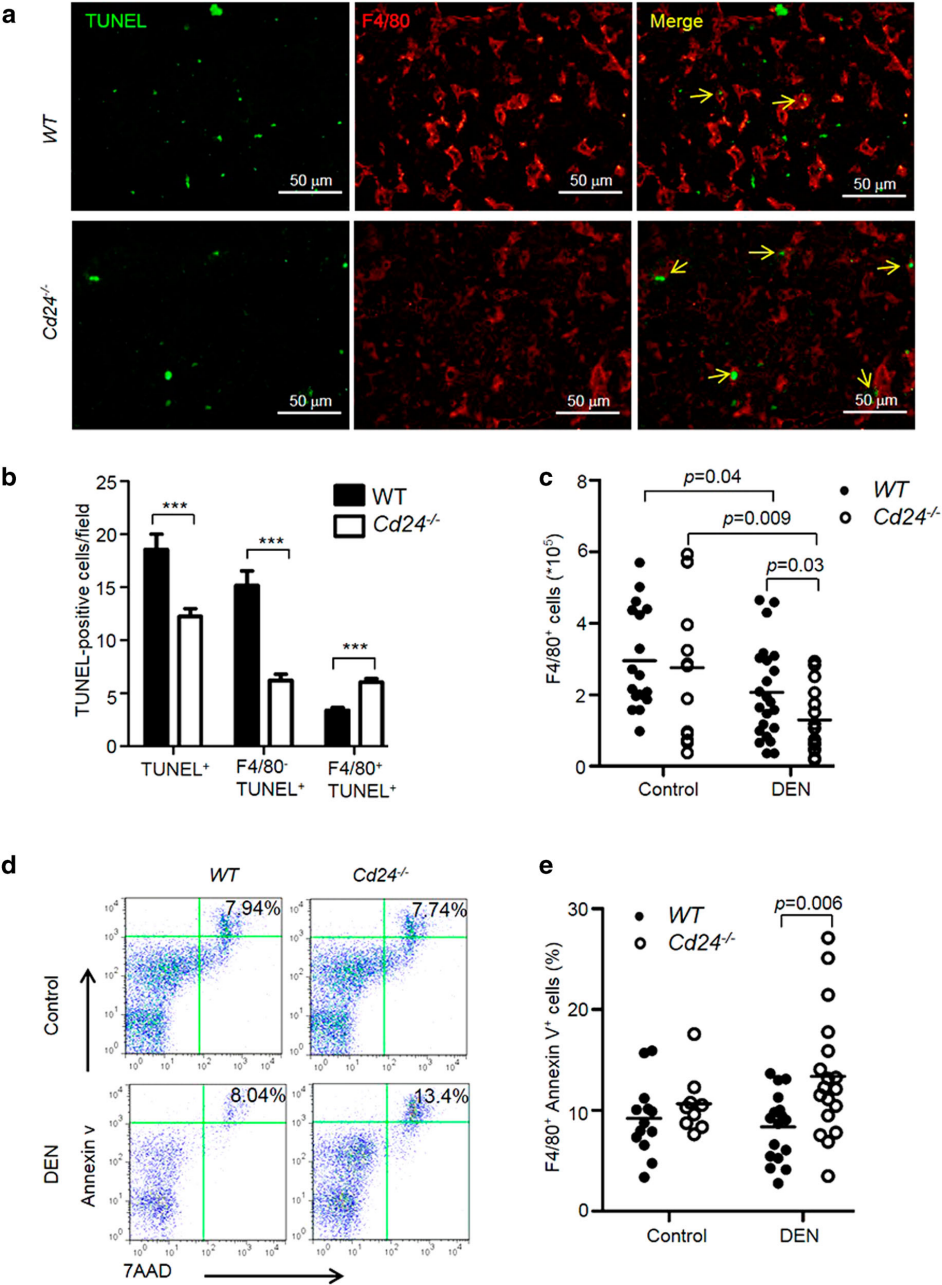
*Cd24* maintains macrophages at acute phase during DEN treatment. **a** and **b** Two-color immunofluorescence staining of frozen liver tissue sections 24 h after mice received 15 μg/g DEN on the 15th day after birth. Representative liver sections stained with anti-F4/80 antibody and TUNEL assay. **a** Apoptotic macrophages (yellow arrows) were F4/80^+^/TUNEL^+^ cells. Scale bar = 100 μm. **b** Average numbers of TUNEL-positive cells in a high-power field of a fluroscerence microscope. At least 10 fields from difference mice were counted. *N* = 9. **c**–**e** Flow cytometry analysis of peritoneal macrophages. 15-day-old mice were treated with 15 μg/g DEN and 24 h later, resident peritoneal cells were harvested by lavage of mouse peritoneal cavity with 3% FCS/PBS. Peritoneal macrophages were stained with F4/80-FITC antibody and PE Annexin V Apoptosis Detection Kit I, and the number and apoptosis of macrophages were analyzed by flow cytometry. **c** DEN selectively reduces the number of peritoneal macrophages in *Cd24*^−/−^ but not WT mice. Data shown are the absolute number of F4/80^+^ cells in the peritoneal cavity at 24 h after DEN treatment. **d** Representative FACS profiles depicting apoptotic macrophages in vehicle or DEN-treated mice. **e** Percentages of F4/80^+^Annexin V^+^ cells in peritoneal cavity. Data are pooled from independent experiments. The data were analyzed by Student’s *t*-test

To test whether apoptosis of intrahepatic macrophages resulted in the decreased number of macrophages in DEN-treated *Cd24*^−/−^ mice, we carried out double staining with anti-F4/80 antibody and TUNEL assay in liver sections. At 24 h after DEN administration, more apoptotic macrophages (F4/80^+^TUNEL^+^ cells) were observed in DEN-treated *Cd24*^−/−^ livers compared with WT livers, in contrast to what was observed with the apoptosis of non-macrophages (F4/80^−^TUNEL^+^ cells) (Fig. 6a, b). To further confirm the increased sensitivity of *Cd24*^−/−^ macrophage to DEN treatment, we measured the apoptosis and number of peritoneal macrophages 24 h after DEN administration. As shown in Fig. 6c, a more severe reduction in the number of peritoneal macrophages was induced by DEN in *Cd24*^−/−^ mice than WT mice. Correspondingly, DEN induced the apoptosis of peritoneal macrophages from *Cd24*^−/−^ but not WT mice (Fig. 6d, e).

**Fig. 7:**
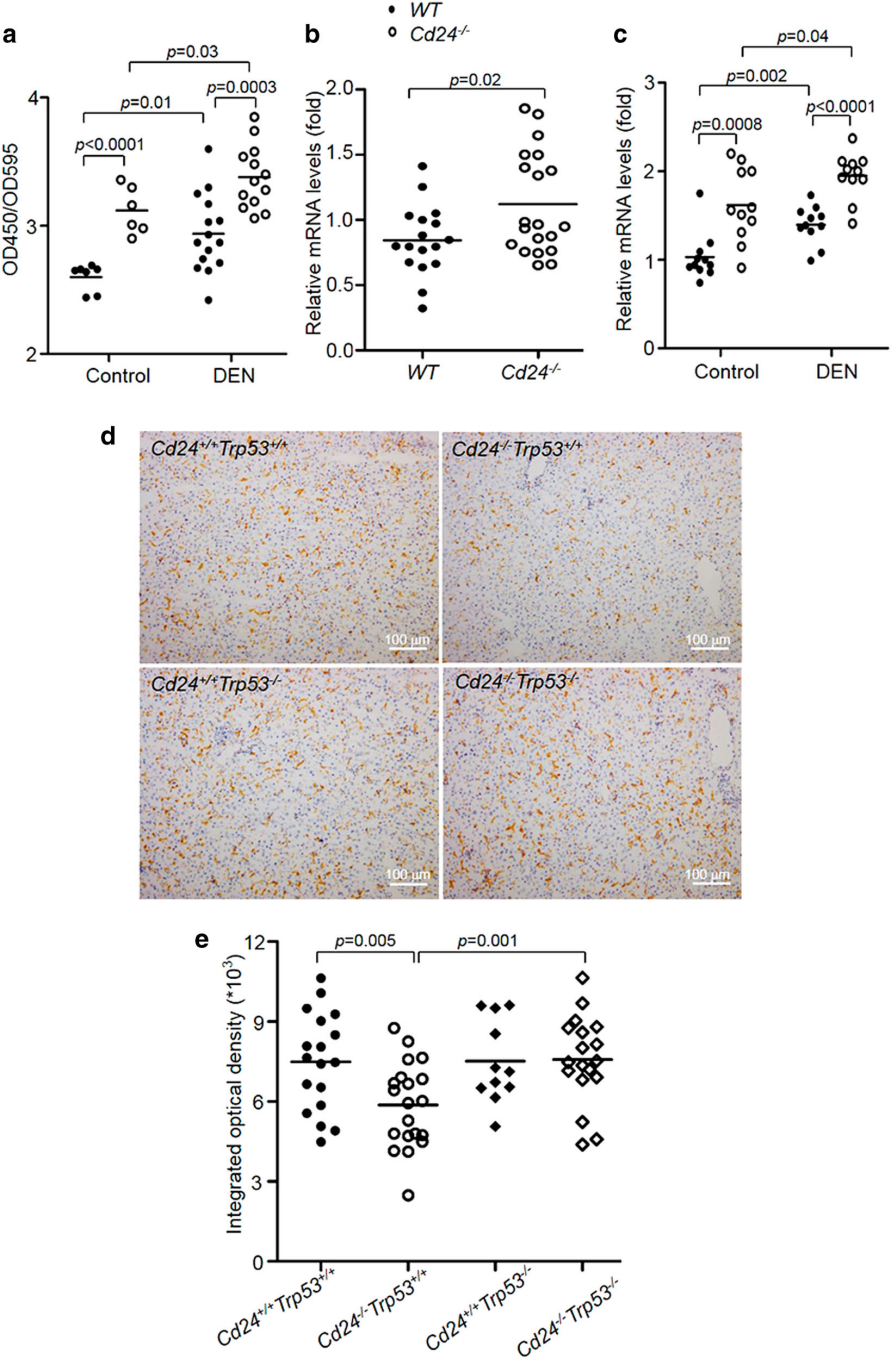
The Cd24–p53 axis sustains macrophages during carcinogenesis. **a** Cell-based ELISA for p53 in macrophages. Macrophages were obtained as described in Figure S11. The p53 protein expression in macrophages was quantitated using a target specific primary antibody and HRP-conjugated secondary antibody detection agent, and the crystal violet provided whole cell staining that was used for cell number counts. The levels of p53 protein were normalized by total cell counts in each well with this formula of OD450/OD595. Each dot represents mean data from one mouse based on triplicate counts. Data from two independent experiments are presented. **b** *Cd24* deficiency increased *p21* mRNA expression after in vivo DEN (15 μg/g) treatment. Data from three independent experiments are pooled. **c** As in **b**, except that WT and *Cd24*^−/−^ macrophages were treated with 20 mM DEN in vitro. Data from two independent experiments are pooled. Statistical significance was determined by student’s *t*-test. **d** F4/80 staining for liver macrophages in DEN-treated mice. *Cd24*^*+/+*^*Trp53*^*+/+*^, *Cd24*^−/−^*Trp53*^*+/+*^, *Cd24*^*+/+*^*Trp53*^−/−^, and *Cd24*^−/−^*Trp53*^−/−^. Scale bar = 100 μm. **e** Integrated optical density of F4/80-positive cells quantified by Image-Pro Plus 6.0 software in a lower-power field of a microscopy. Twenty fields in each section were counted. Data from multiple experiments are presented. Statistical significances were analyzed by Student’s *t*-test

DEN is a typical chemical carcinogen that causes DNA damage and mutations once activated by cytochrome P450 2E1^29^. DNA damage induces the activation of p53 pathway to eliminate damaged cells^30^. Our recent publication showed that Cd24 disrupts ARF-NPM association and reduces cellular p53 levels in cancer cells^47^. We therefore analyzed Cd24 expression on liver cells, and found Cd24 was expressed on hematopoietic cells, but not hepatocytes or tumor cells of HCC and naive mice (Supplementary Figure S10A and B). Flow cytometry analysis revealed high levels of Cd24 on intrahepatic macrophages as well as other leukocytes (Supplementary Figure S10C and D), which raised the intriguing possibility that the CD24–p53 antagonism discovered in cancer cells may regulate macrophage survival. In order to determine whether Cd24 regulates p53 in macrophages, we examined the expression of p53 and p53-regulated genes in peritoneal macrophages following DEN treatment. The purity of macrophages after isolation was >90% based on immunofluorescence microscopy result (Supplementary Figure S11A). As shown in Fig. 7a, p53 protein levels were elevated in both WT and *Cd24*^−/−^ macrophages after DEN treatment. However, *Cd24*^−/−^ macrophages expressed more p53 protein than WT macrophages regardless DEN treatment (Fig. 7a). To determine the functional consequence of p53 upregulation, we measured the transcript levels of *p21* and *puma*, the prototypic target gene of p53, in DEN-treated macrophages. As shown in Fig. 7b, mRNA level of *p21* was significantly higher in *Cd24*^−/−^ macrophages, whereas no obvious difference was found in *puma* expression between WT and *Cd24*^−/−^ macrophages (Supplementary Figure S11B). To substantiate this observation, macrophages were isolated from naive WT and *Cd24*^−/−^ mice and then treated with DEN in vitro for 24 h. As shown in Fig. 7c, *Cd24*^−/−^ macrophages also exhibited higher levels of *p21* mRNA at 24 h after DEN administration relative to WT macrophages that received the same treatment. However, the mRNA levels of *puma* were not changed after DEN administration, and no difference was observed between WT and *Cd24*^−/−^ mice (Supplementary Figure S11C). These data suggest that the effector that p53 target for apoptosis in macrophages is unlikely Puma. As p21 is involved in senescence rather than apoptosis, the p53 responsive target responsible for apoptosis remain to be defined in this model.

To determine whether elevation of p53 is responsible for apoptosis of macrophages, we produced *Cd24*^−/−^ *Trp53*^−/−^, *Cd24*^*+/+*^*Trp53*^−/−^, *Cd24*^−/−^*Trp53*^*+/+*^*,Cd24*^*+/+*^*Trp53*^*+/+*^ mice by intercrossing mice with deletion of either gene. We then treated 15-day-old male mice with 15 μg/g DEN. Twenty-four hours later, intrahepatic macrophages were assessed by IHC with anti-F4/80 antibody. Images of representative slides from one mouse in each group are shown in Fig. 7d, whereas summary data from 18 pairs of mice were summarized in Fig. 7e. As expected, at 24 h after DEN injection, intrahepatic F4/80^+^ cells were significantly reduced in *Cd24*^−/−^*Trp53*^*+/+*^ mice when compared with *Cd24*^*+/+*^*Trp53*^*+/+*^ mice. Remarkably, the impact of Cd24 was completely ablated with *Trp53* deletion (*Cd24*^*+/+*^*Trp53*^−/−^). Thus, loss of intrahepatic macrophages after DEN treatment of the *Cd24*^−/−^ mice is attributed to the upregulation of *Trp53* gene.

## Discussion

Based on the current paradigm that inflammation promotes HCC carcinogenesis and the fact that carcinogen DEN induces extensive death of hepatocytes^5^, we evaluated the impact of targeted mutation of *Cd24*, which exacerbates host response to cellular injuries^38,48^. Surprisingly, whereas *Cd24* deletion did increase susceptibility to HCC, it reduced rather than increased inflammatory response to DEN. Our in-depth analysis of this model revealed new insights on the function of Cd24 in macrophages and the function of intrahepatic macrophages on HCC pathogenesis.

Although a large body of circumstantial evidence is consistent with the notion that intrahepatic macrophages promote HCC development, the proposed cancer-promoting function of macrophages has not been established by either macrophage depletion or macrophage-specific gene deletion. Here, we provide two lines of direct evidence that argue against this prevailing view.

First, macrophages are responsible for the apoptosis of DEN-treated hepatocytes in vivo, some of which harbor cancer-causing mutations. This function is suggested by the correlation between the amounts of intrahepatic macrophages and the amounts of hepatocyte apoptosis and, more importantly, validated by in vivo depletion of macrophages. As the depletion of macrophages substantially reduced hepatocyte apoptosis in DEN-treated WT mice, our data demonstrate a role for macrophages in inducing hepatocyte apoptosis. Considering DEN is a known DNA damaging agent^29^, we suggest that during acute inflammation, macrophages may reduce HCC risk by eliminating cells with DNA damage. A similar function has been suggested for NK cells by in vitro studies^31^. Furthermore, macrophages have been shown to clear precancerous senescent hepatocytes^49^. In addition, as removing macrophages in the *Cd24-*deficient mice does not affect the amounts of apoptotic cells, it is likely that Cd24 deficiency has ablated the activity of intrahepatic macrophages, either through functional inactivation or through reduction in the number of intrahepatic macrophages.

Second, depletion of macrophages at DEN induction, or 5 months after, substantially increased HCC carcinogenesis. Although the impact of macrophage depletion at DEN induction suggests that removing cells with DNA damage will likely reduce the chance of carcinogenesis, the dramatic impact of macrophage depletion at five months after DEN induction argues against the notion that macrophages evolve into HCC-promoting M2-type macrophages.

Although the focus of the current study was to establish the functional significance of intrahepatic macrophages rather than the mechanism of its effector function, our data suggest some potential mechanisms for future studies. We showed that WT but not *Cd24*^−/−^ mice had high levels of ROS 1 day after DEN treatment. As the ROS^+^ cells are mononuclear cells rather than hepatocytes, and ROS are known to be cytotoxic for DEN-treated hepatocytes, we suggest that elevation of ROS in WT mice (as compared with *Cd24*^−/−^ mice) provides an effector mechanism for intrahepatic macrophages. The reduction of ROS^+^ cells also explains the reduced hepatocyte apoptosis in the *Cd24*^−/−^ mice. Consistent with this notion, we found that pre-treatment with butylated BHA significantly reduced hepatocyte apoptosis in WT but not *Cd24*^−/−^ mice. Alternatively, we have observed much higher levels of iNOS among intrahepatic macrophages in WT mice when compared with *Cd24*^−/−^ mice. As NO is a known effector molecule produced by macrophages^50,51^, the increase in iNOS not only provides another mechanism by which intrahepatic macrophages induce apoptosis of hepatocytes during DEN treatment, but also suggests a possible role for *Cd24* in the differentiation of macrophages.

It should be pointed out that the surveillance function of macrophages may be achieved by different mechanisms at different stages of carcinogenesis. Although our data suggested that in the early stage, macrophages help to remove hepatocytes with DNA damage, how the macrophages that surround HCC lesions restrict their growth remains to be investigated.

As outlined in the Introduction, the evidence that supports the role for TAM in HCC human carcinogenesis is based on their ability to either directly suppress CD4 T-cell responses or indirectly induce Tregs that in turn would reduce adaptive T-cell immunity^52^. It therefore remains possible that when T-cell response is the key determinant, macrophages may play a suppressive function. Alternatively, apart from the immune suppressive features, the TAM in human HCC may also possess an innate protective function as described herein. The ultimate function of TAM would depend on the balance of these two opposing forces. In the mouse DEN model, however, all genetic studies supporting a role for macrophages in promoting tumor growth have used drivers that cause deletion in all leukocytes and non-leukocytes^19,20^. Therefore, these studies do not allow comparison with our data presented herein. Much like other studies cited herein, our model depends on one-time exposure to a genotoxin and thus may differ somewhat from human HCC carcinogenesis that likely results from chronic exposure to toxic chemicals or chronic viral infection. However, our work is more decisive in establishing a surveillance function or macrophages in the DEN-induced HCC model because we showed its function by direct depletion. The relevance of *Cd24* in human HCC pathogenesis is supported by the fact that *Cd24* polymorphism affects the risk and progression of HCC^53,54^.

Based on our recent studies in cancer cells, in which we showed that *Cd24* increases p53 levels through antagonism with the ARF-MDM2 pathway^47^, we evaluated the role for p53 in macrophages survival. Our genetic studies clearly revealed that *Trp53* is downstream of *Cd24* in regulating survival of intrahepatic macrophages. Our genetic evidence provides a new concept that tumor suppressor gene could regulate host immunity through its impact on the survival of innate immune effectors. Apart from the macrophage-intrinsic protective function, a large body of evidence shows a costimulatory function of CD24 for T-cell activation^34,36,55,56^. Therefore in models in which CD4 T cells promote cancer development, one may expect a different consequence of targeted mutation of the *Cd24* gene. In this context, it would be of great interest to reconcile the cancer-promoting function of the *Cd24* gene in HCC caused by a HBV transgene, as we have reported^54^. Furthermore, given the expression of CD24 on B cells and a potential role for B cells in HCC pathogenesis^57^, it is of interest to investigate the function of CD24 on B cells during HCC pathogenesis in the future study.

## Materials and methods

### Experimental animals

Mice with the targeted mutation of *Cd24* were produced using embryonic stem cells from C57BL/6 mice as described previously^39^. *Trp53* mutant mice B6.129S2-Trp53^tm1Tyj^/JNju (referred to as *Trp53*^*+/−*^ mice)^58^ were purchased from Nanjing Biomedical Research Institute of Nanjing University. *Trp53*^*+/−*^ mice were crossed with *Cd24*^−/−^ mice, and the offsprings were genotyped by genomic polymerase chain reaction (PCR) to get the *Cd24*^*+/*−^*Trp53*^*+/−*^ mice. Then *Cd24*^*+/−*^*Trp53*^*+/−*^ mice were intercrossed to generate *Cd24*^*+/+*^*Trp53*^−/−^, *Cd24*^−/−^ *Trp53*^−/−^, *Cd24*^*+/+*^*Trp53*^*+/+*^, and *Cd24*^−/−^*Trp53*^*+/+*^ mice. *Cd24*^−/−^ and WT mice on a C57BL/6 background were intercrossed to generate *Cd24*^−/−^ mice and WT littermates. The genotypes were determined by PCR. All of the mice were maintained under specific pathogen-free conditions in the animal facility at the Institute of Biophysics, Chinese Academy of Sciences. All studies involving animals were approved by the Institutional Laboratory Animal Care and Use Committee.

### Induction of HCC

Fifteen-day-old male mice were injected intraperitoneally (i.p.) with 15 μg/g DEN (Sigma), as indicated. For the tumor initiation model, after 1, 4, 7, and 8 months on normal chow, all mice were killed, and their livers were removed. Externally visible tumors (≥1.0 mm) were counted. The length (*L*) and width (*W*) of tumors were measured by a vernier caliper. The tumor volume was calculated by (*L*×*W*×*W*)/2. For the early effects of DEN, mice were killed 24 h after DEN treatment and peritoneal resident macrophages and liver tissues were collected. The liver tissues were stored at −80 °C for protein isolation, TRizol reagent (Invitrogen) for RNA extract, and embedded in optimal cutting temperature compound (OCT) or fixed in 10% formalin for histological staining. The remaining liver tissues were used for isolation of liver mononuclear cells.

### Histology and immunofluorescence

The large lobes of livers were immediately embedded in OCT, or fixed in 10% formalin and paraffin-embedded. Sections (5.0 μm) were subjected to analysis of histological and immunochemical staining.

To examine the accumulation of ROS, freshly prepared frozen liver sections were incubated with 2.0 μM dihydroethidine (Invitrogen) for 30 min at 37 °C, after which they were observed by fluorescence microscopy and photographed.

TUNEL assays were performed using the In Situ Cell Death Detection Kit (Roche) on paraffin-embedded liver sections. For double immunofluorescence staining of F4/80 and TUNEL, frozen sections were stained with F4/80 antibody, followed by permeabilization with 1% citrate for 15 min at 4 °C, and then TUNEL staining was performed according to the manufacturer’s instructions. The mounted slides were observed under a microscope (Leica DM2500). The number of TUNEL-positive cells was determined by manual counting of at least 5 high-power fields per section.

The immunostaining on frozen liver sections was performed as previously described^54^. Primary antibodies were rat anti-F4/80 (Abcam, ab6640, 1/100), rabbit anti-F4/80 (Santa Cruz, sc-25830, 1/50), rat anti-CD24 (Santa Cruz, sc-19651, 1/100), rabbit p-Histone H2AX (γ-H2AX) (Bioworld Technology, Inc., BS4760, 1/100), goat anti-HNF4α (Santa Cruz, sc-6556, 1/50), rabbit anti-PCNA (Santa Cruz, sc-7907, 1/300). Secondary antibodies were purchased from Santa Cruz and Zhongshan Goldenbridge Biotech (Beijing, China). For microscope images, positive cells were quantified with Image-Pro Plus 6.0 software (Media Cybernetics, Inc.).

### Cell isolation

For isolation of liver mononuclear cells, the liver fragments were incubated for 30 min in 0.025% collagenase type IV in HBSS (Gibco) at 37 °C shaking at 220 rpm. The whole material was passed through a 70 μm cell strainer to get a single cell suspension, centrifuged with 50 G for 3 min to remove hepatocytes. The liver mononuclear cells were recovered from the interface of 40 and 60% percoll (GE Healthcare) after centrifugation. The liver mononuclear cells were washed and used for flow cytometry. To enrich macrophages, mononuclear cells were suspended in serum-free RPMI-1640 medium and were seeded into 24-well plate. After incubation for 2 h, the cells were washed three times with PBS to remove the non-adherent cells. The adherent cells were used as source for intrahepatic macrophages, and the purity of macrophages was determined by morphology and immunofluorescence staining with F4/80 antibody.

Peritoneal resident cells were harvested by lavage of mouse peritoneal cavity with 2.0 mL of ice cold 3% FCS/PBS according to the protocol as described^59^. The collected cells were centrifuged at 1300 rpm for 10 min, and re-suspended in RPMI-1640 medium or PBS for counting. The cells in RPMI-1640 medium were seeded into the wells of 24-well or 96-well plate. After incubation for 2 h, the cells were washed three times with PBS to remove the non-adherent cells. The adherent cells were macrophages for cell ELISA (for p53) and RT-PCR (for *p21* and *puma*), or were treated with 20 mM DEN for 24 h for RNA extract (for *p21* and *puma*). For peritoneal macrophages by flow cytometry, the re-suspended cells in PBS were immediately counted, washed twice in PBS, and re-suspended in FACS buffer (2% FBS in PBS containing 0.01% NaN3).

### Flow cytometry

The following antibodies were used for FACS: FITC-anti-CD11b (11–0112), APC-anti-CD11b (17–0112), PE-anti-F4/80 (12–4801), FITC-anti-F4/80 (11–4801), APC-anti-CD45 (17–0451), PerCp-Cy5.5-anti-CD45 (45–0451), FITC-anti-CD24 (11–0242), APC-anti-CD24 (17–0242), PerCP-Cy5.5-anti-NK1.1 (45–5941), PE-anti-CD4 (12–0041), FITC-CD3e (11–0031), PerCP-Cy5.5-Gr1 (45–5931), and PE-anti-Ly6C (12–5932) obtained from eBioScience, and PE Annexin V Apoptosis Detection Kit I (559763) from BD Pharmingen. Cells were blocked for 15 min with Fc blocking reagent on ice prior to labeled with fluorescent-conjugated antibodies diluted in FACS buffer. Cells for phenotypic analyses were stained using the indicated fluorescent-conjugated antibody for 30 min on ice. Appropriate isotype controls were used in all case. Flow cytometry was performed using a FACSCalibur (BD Bioscience, San Jose, CA) and the data were analyzed with FlowJo software (TreeStar, Ashland, OR).

### Macrophage depletion

To deplete macrophages,13-day-old mice were injected i.p. with 70 μl of liposomal clodronate (5 mg/ml clodronate) and liposomal control (no clodronate) (Encapsula NanoSciences LLC). Fourty-eight hours later, the mice were treated with DEN. In some experiments, mice were killed 24 h after DEN administration for analysis of apoptosis. For the tumor initiation model, mice were kept for additional 5 months, after which they were killed and analyzed to determine tumor load. Depletion of liver macrophages was assessed 72 h after clodronate administration by immunohistochemical staining of frozen liver sections with F4/80 antibody, and RT-PCR analysis of F4/80 mRNA levels.

To deplete liver macrophages after tumor already formed, WT mice were injected with 15 μg/g DEN at 15-day of ages. After 5 months, 200 μL of 1:1 PBS-diluted liposomes were injected via the vail vein once every 5 days for 3 times. All mice were killed 1 month after the last injection.

### RNA extraction and RT-PCR

Total RNA was isolated from adherent peritoneal or liver macrophages using the miRNeasy Mini Kit (Qiagen) according to the manufacturer’s protocol. cDNA was synthesized using total RNA via PrimeScript RT reagent Kit with gDNA Eraser (Takana) according to the manufacturer’s instructions. Real-time PCR was performed using SYBR green PCR master mix (Applied Biosystem, USA) in a 7500 Real-Time PCR System (ABI). Expression levels of evaluated genes were calculated with the comparative ∆Ct method using hypoxanthine guanine phosphoribosyl transferase as a reference gene, and relative abundance was quantified with the 2^-∆∆Ct^ method. The following forward and reverse specific primer sequences were used: *Il1b*-F (TGAAGCAGCTATGGCAACTG), *Il1b*-R (AGGTCAAAGGTTTG GAAGCA); *Il-6*-F (GAGGATACCACTCCCAACAGACC), *Il-6*-R (AAGTGCATCA TCGTTGTTCATACA); *Tnfa*-F (CAGCCTCTTCTCATTCCTGC), *Tnfa*-R (GGTC TGGGCCATAGAACTGA); *Mcp-1*-F (TGCATCTGCCCTAAGGTCTTC), *Mcp-1*-R (AAGTGCTTGAGGTGGTTGTGG); *p21*-F (CGAGAACGGTGGAACTTTGACT), *p21*-R (GGACCCAGGGCTCAGGTAGAC); *puma*-F (ACCTCAACGCGCAGTACGA), *puma*-R (GGGAGGAGTCCCATGAAGAGA); *Emr1*-F (ACTGTGGAAAGCACCATG TTAGC), *Emr1*-R (TTTCGATGTCTAGGTACTCCGTC); *iNOS*-F (TGAAGAAA ACCCCTTGTGCT), *iNOS*-R (TTCTGTGCTGTCCCAGTGAG); *Hprt*-F (ATGAG CGCAAGTTGAATCTG), *Hprt*-R (CAGATGGCC ACAGGACTAGA).

### Cell-based ELISA for p53 in macrophages

The p53 protein expression in macrophages was quantitatively determined using Total p53 Cell-Based Colorimetric ELISA Kit (ImmunoWay, USA) according to the manufacturer’s instructions. In brief, isolated macrophages in a 96-well plate in triplicate were fixed in 4% formaldehyde for 30 min. Each well was incubated with anti-p53 antibody overnight at 4 °C. After washing three times with wash buffer, samples were incubated with HRP-conjugated secondary antibody followed by incubation with TMB substrate. Read the absorbance at 450 nm after adding stop solution. Crystal violet binds to cell nuclei and gives absorbance reading at 595 nm that is proportional to cell numbers. After washing the plate several times, each well was stained with the crystal violet solution, after which the absorbance at 595 nm was measured. The levels of p53 protein were normalized by total cell number in each well with this formula of OD450/OD595.

### Protein preparation and western blot

Total proteins were isolated from frozen liver tissues after homogenization in RIPA buffer supplemented with proteinase inhibitor protease (cocktail and PMFS) and phosphatase inhibitors (Na_3_VO_4_, BETA-glycerophosphate and Na_2_P_2_O_4_) on ice. Protein from tissue lysate was quantified using the *DC* Protein Assay Kit (Bio-Rad). Equal amounts of protein were used for western blots and cytokine measurement. Western blot was performed according to standard protocols. Lysed proteins were separated using 10% SDS/PAGE and transferred to polyvinylidene difluoride membranes (Millipore). Membranes were exposed to antibodies that recognized STAT3 (CST, no. 4904), P-STAT3 (CST, no. 9145), and β-actin (Bioworld, no. AP0060). The β-actin was used as an internal control to normalize the loading materials.

### Cytokine determination

Cytokines were measured from serum and liver lysate using Mouse Enhanced Sensitivity Flex Set from BD Cytometric Bead Array (CBA, BD Biosciences) according to the manufacturer’s instructions. CBA for IL-1β, IL-6, TNF, IL-17A, and IFN-γ were carried out, and data were acquired on FACS AriaII (BD Bioscience, San Jose, CA). Serum levels of HMGB1 were measured using commercial kits (BIOPCR, H20734) according to the manufacturer’s protocol.

### Statistical analysis

The differences in mean values between groups were analyzed by a two-tailed unpaired Independent-Samples *t*-test unless otherwise indicated. For tumor volume comparison, the total tumor volumes in mice were presented as a logarithmic distribution. After the log10 transformation, the volumes for different subgroups were presented in a normal distribution (*p* > 0.05 in a One-Sample Kolmogorov–Smirnov test). Then, the difference in mean log volumes between mice with WT and *Cd24*^−/−^ genotypes was analyzed by Student’s *t*-test. These statistical analyses were performed with the GraphPad Prism 5 Software (GraphPad, San Diego, CA) and SPSS19.0 Software (SPSS, Chicago, USA). Data are presented as means or means ± SEM. A result was considered significant if a *p*-value was <0.05 (*p < 0.05, **p < 0.01, ***p < 0.001).

## Electronic supplementary material

Supplementary Information
