# Supplementary material for: CD24–p53 axis suppresses diethylnitrosamine-induced hepatocellular carcinogenesis by sustaining intrahepatic macrophages
Source: Cell Discov. 2018 Feb 6;4:6. doi: 10.1038/s41421-017-0007-9 (PMC5799181; doi:10.1038/s41421-017-0007-9)
Supplement: Supplementary file 1 — Supplementary Information [file 41421_2017_7_MOESM1_ESM.pdf]

Supplementary Figures

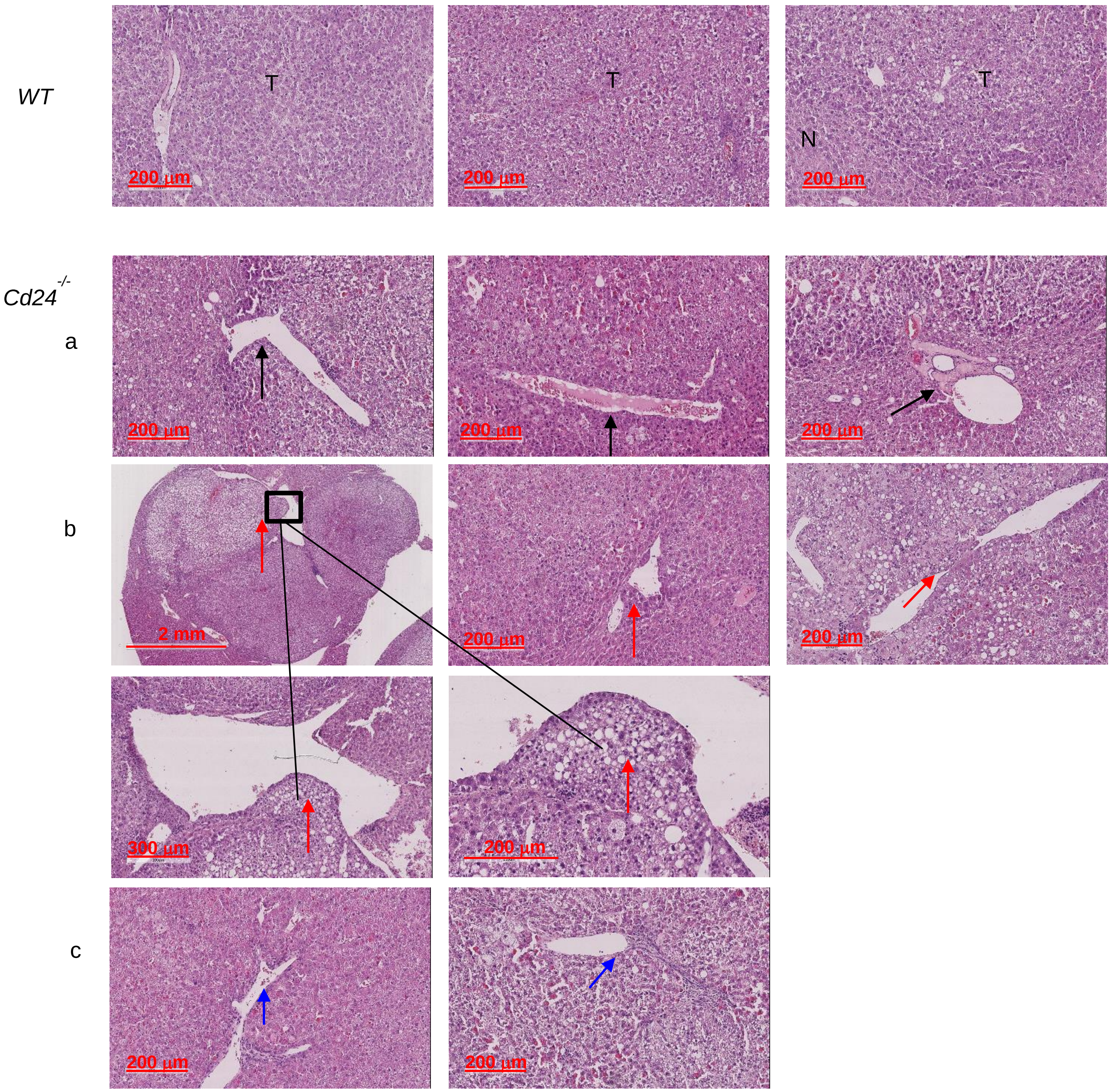

Figure S1. Related to Figure 1. At 8 months post induction, DEN-treated *Cd24*$^{-/-}$ mice developed HCC, but lesions (T) and nodules in *WT* mice are either precancerous lesions or low grade adenomas. Representative HCC features from *Cd24*$^{-/-}$ mice are shown in a-c. (a) Ductules extended to the peripheries of foci lesions, and associated with bile ducts (black arrow). (b) Foci lesions penetrated the walls and proliferated in the lumens of a hepatic vein branch (red arrow). (c) Vascularization by portal vein branches (blue arrow).

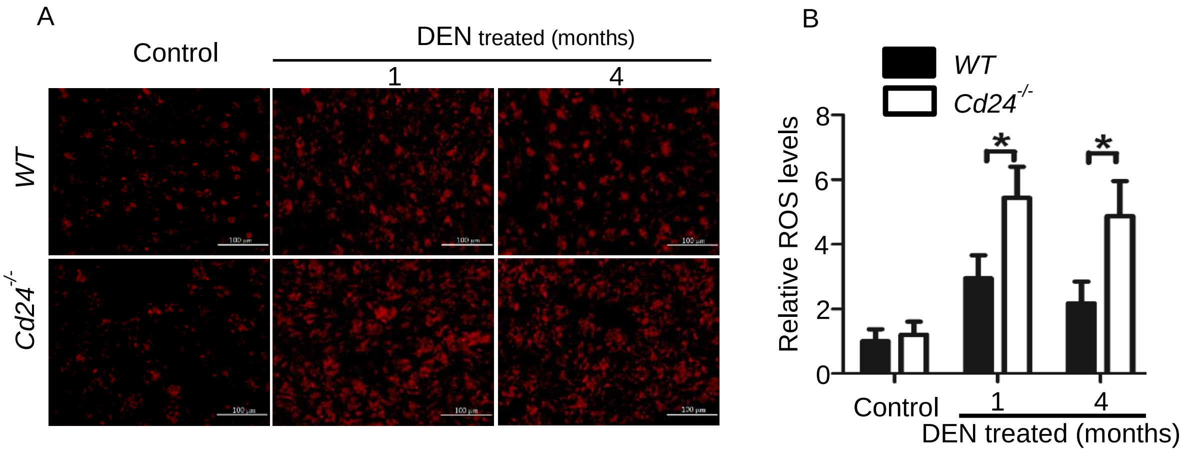

Figure S2. Related to Figure 2. At one or four months after the DEN treatment, ROS-producing cells had the morphology of hepatocytes and were increased in the *Cd24*$^{-/-}$ mice. Liver cryosections prepared at the indicated time points were incubated with 2 μM dihydroethidine for 30 min at 37°C. Cells staining positive for the oxidized dye (red) were identified by fluorescence microscopy. (A) Representative images of ROS staining in control or DEN-treated liver sections. Scale bar =100 μm. (B) Quantitative analysis or ROS levels using Image-Pro Plus 6.0 software. At least 10 fields from each mouse were counted. Data shown are summarized from 5 control and 12 DEN-treated mice.

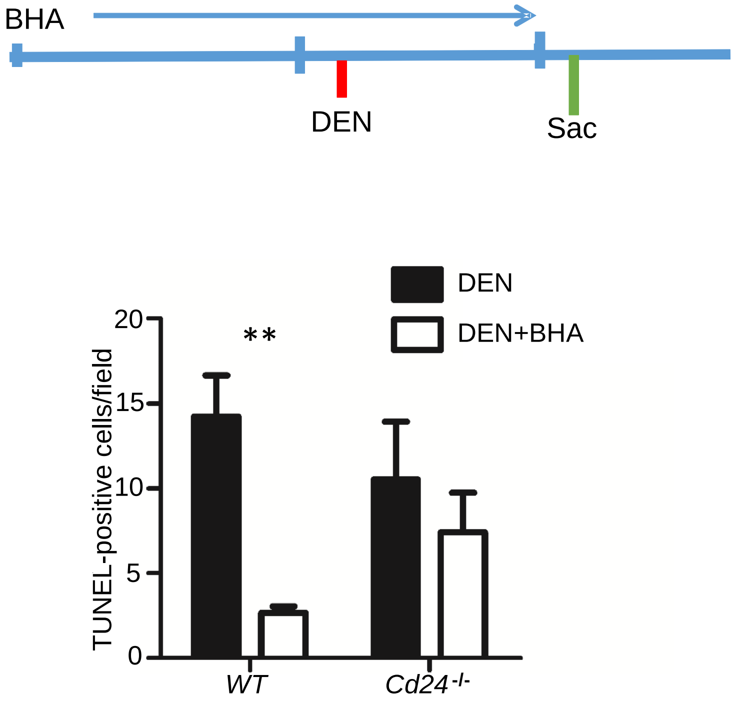

Figure S3. Numbers of TUNEL-positive hepatocytes at 24 hours after DEN treatment, related to Figure 2. Mice of 6-8 wks old were administrated with 300 μg/g butylated hydroxyanisole (BHA) by oral gavage once a day for three days, as indicated by vertical blue bar. At 24 hours after the BHA treatment, the mice were treated with 15 μg /g of DEN or vehicle. Mice were sacrificed at 24 hours after DEN treatment DEN mice: n=5; DEN+BHA mice, n=6.

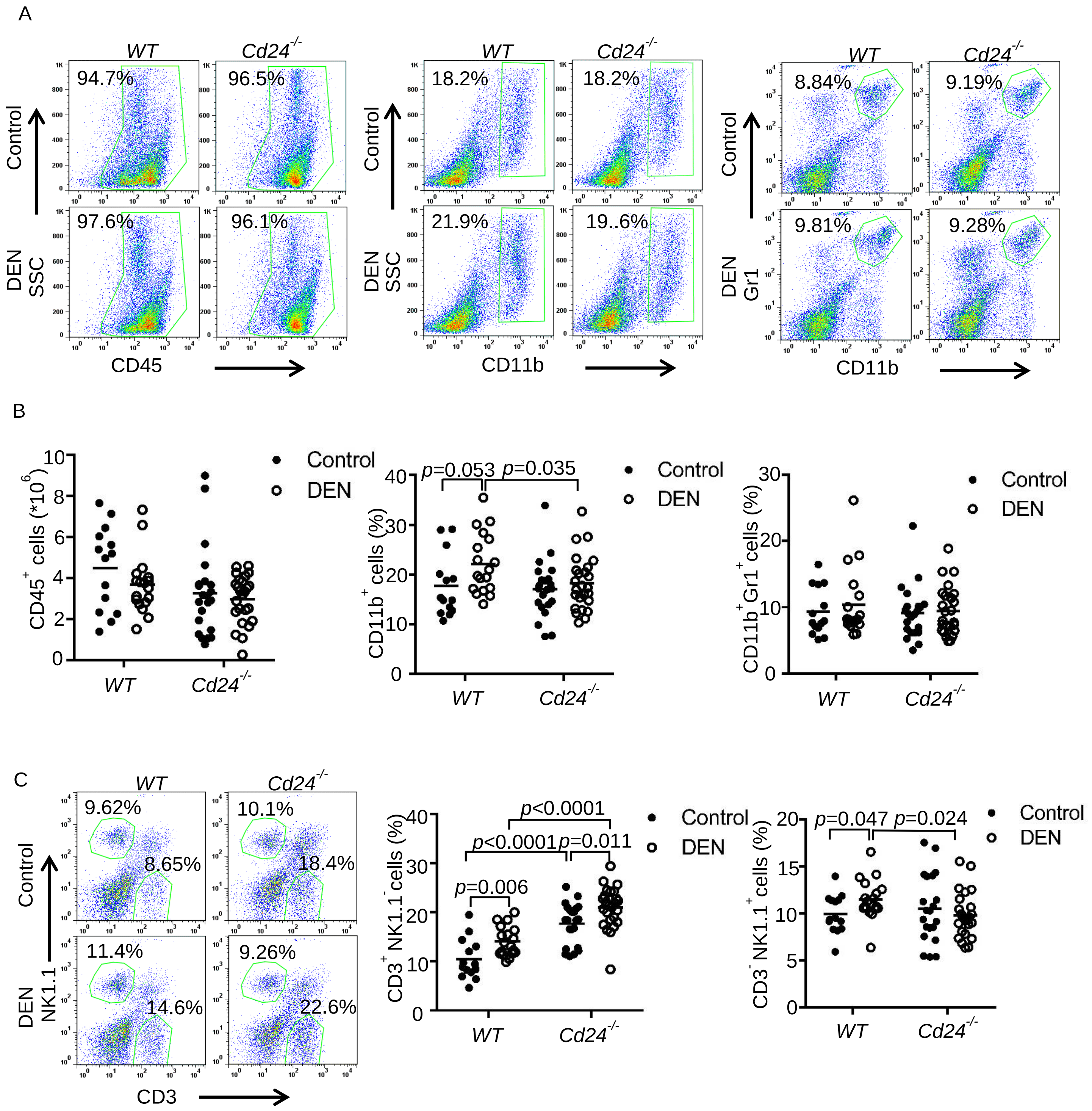
A
WT
Cd24-/-
94.7%
96.5%
97.6%
96.1%
Control
DEN
SSC
CD45
18.2%
18.2%
21.9%
19..6%
CD11b
8.84%
9.19%
9.81%
9.28%
Gr1
B
CD45+ cells (*10^6)
Control
DEN
CD11b+ cells (%)
p=0.053
p=0.035
CD11b+Gr1+ cells (%)
C
9.62%
10.1%
8.65%
18.4%
11.4%
9.26%
14.6%
22.6%
NK1.1
CD3
CD3+ NK1.1- cells (%)
p<0.0001
p<0.0001
p=0.011
p=0.006
CD3- NK1.1+ cells (%)
p=0.047
p=0.024

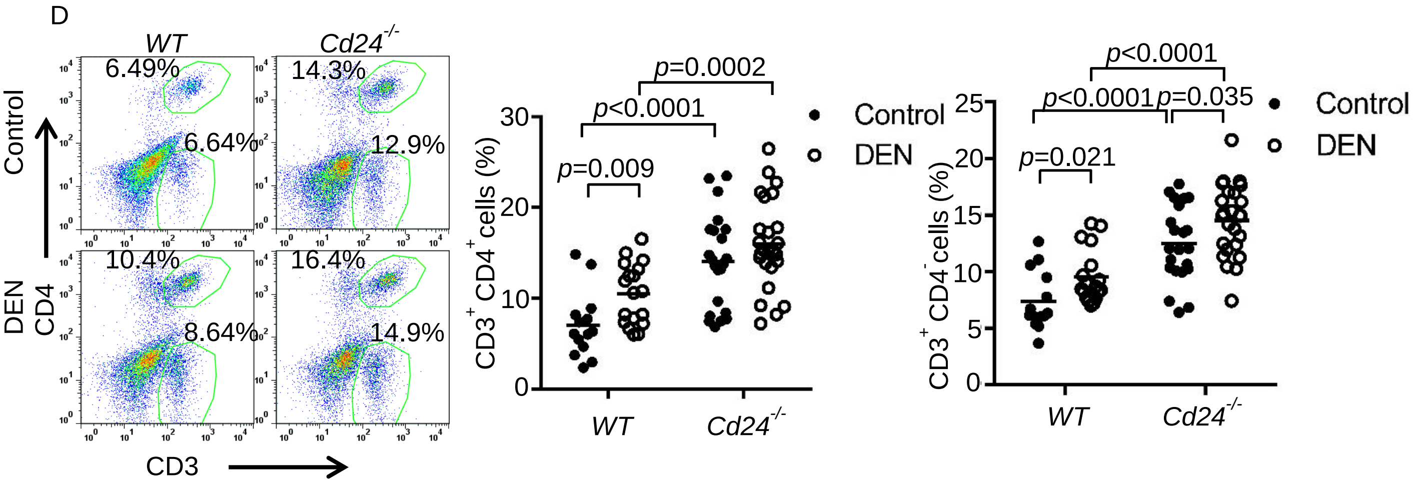

Figure S4. Related to Figure 3. Flow cytometry analysis of liver immune cells. Liver mononuclear cells were isolated 24 hrs after 15 μg/g DEN or 0.9% NaCl solution (as control) injection to 15-day-old mice for FACS. (A) Representative FACS profiles for different intrahepatic immune cell populations, pre-gated on CD45 (CD11b$^+$ cells and neutrophils). (B) Number of leukocytes (CD45$^+$ cells) and percentages of CD11b$^+$ cells and neutrophils among CD45$^+$ cells in liver. (C) Representative FACS profiles and percentages of T cells and NK cells, pre-gated on CD45. (D) Representative FACS profiles and percentages of T cells, pre-gated on CD45$^+$NK1.1$^-$. Data are pooled from multiple experiments involving age and gender matching mice. The data were analyzed by Student's *t* test. Data are means.

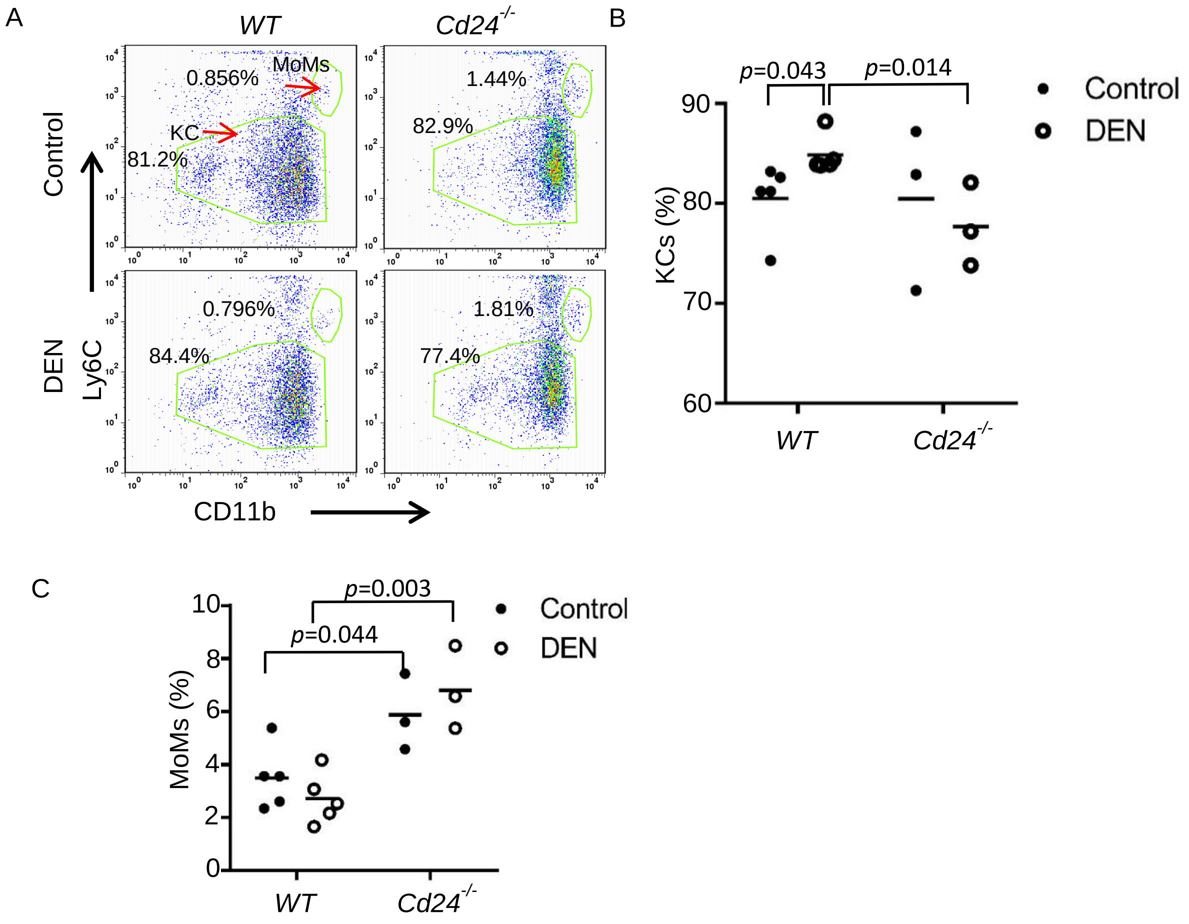

Figure S5. Related to Figure 3. Flow cytometry analysis of intrahepatic F4/80$^+$ cells. Liver mononuclear cells were isolated 24 hrs after 15 μg/g DEN or 0.9% NaCl solution (as control) injection to 15-day-old mice for FACS. (A) The data are representative FACS profiles, pre-gated on CD45$^+$F4/80$^+$ cells. F4/80$^+$CD11b^{lo}Ly6C^{lo} cells were defined as liver-resident kupffer cells (KCs), and F4/80$^+$CD11b^{hi}Ly6C^{int} cells were defined as monocyte-derived macrophages (MoMs). (B) Percentages of KCs and MoMs among CD45$^+$F4/80$^+$ cells in liver. Data represent the representative experiments from 2 independent experiments. The data were analyzed by Student's *t* test. Data are values of individual mice with means shown as black bars.

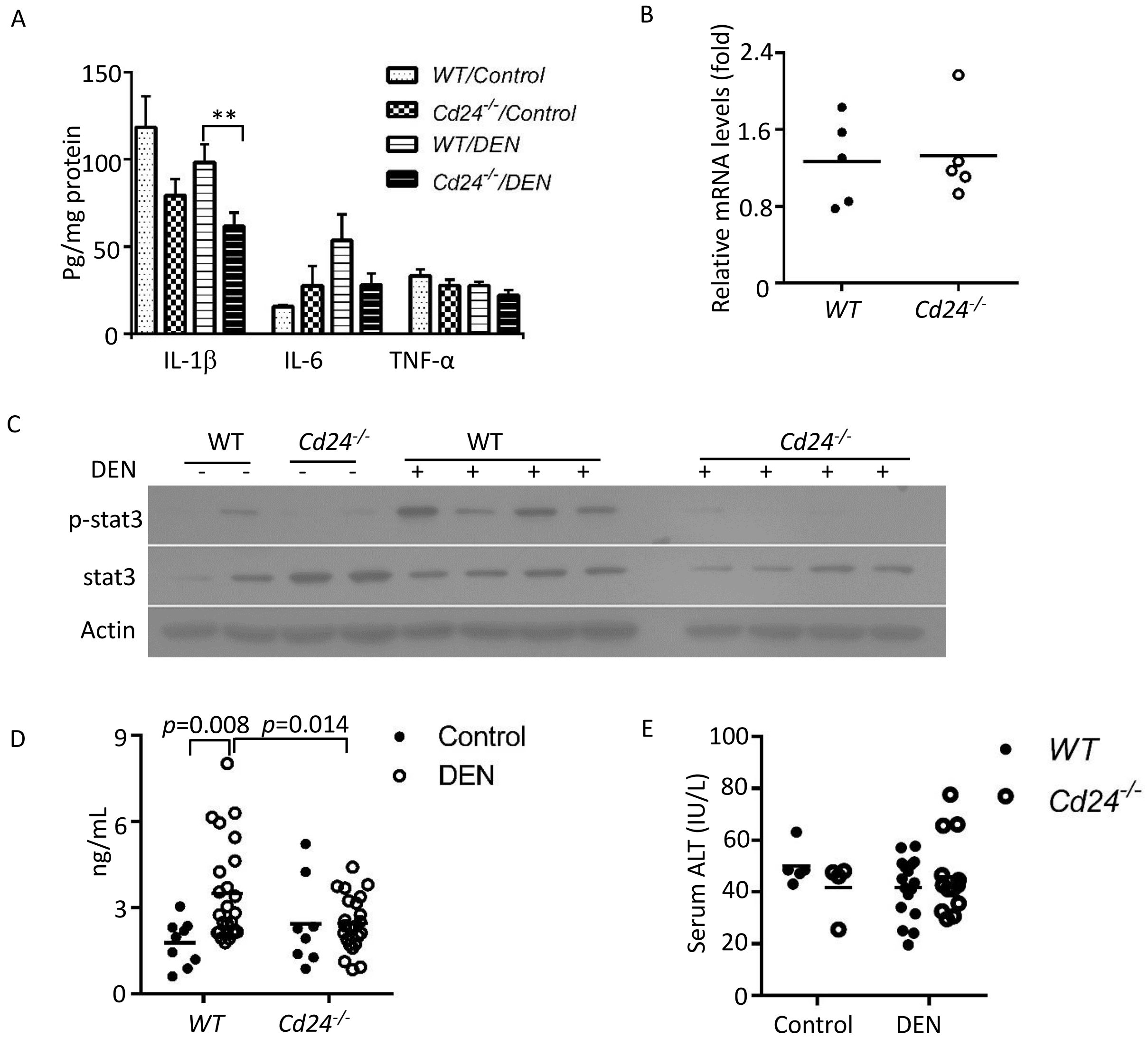

Figure S6. Related to Figure 3. (A) The protein levels of some cytokines were determined by BD CBA Mouse Enhanced Sensitivity Flex Set. Liver tissues were isolated 24 hrs after 15 μg/g DEN injection to 15-day-old mice, and total proteins were extracted for cytokine measure. (Control: n=5; DEN-mice: n≥11). (B) The mRNA levels of *Mcp-1* were

determined by Real-time PCR. Liver tissues were isolated 24 hrs after 100 μg/g DEN injection to 15-day-old mice for measure. (C). STAT3 phosphorylation in liver tissues 24 hrs after 15 μg/g DEN injection to 15-day-old of mice. Lysates of liver tissues from untreated (-) or DEN-treated (+) mice were gel separated and analyzed by immunoblotting. Control: n=4; DEN-mice: n=8). (D). Serum HMGB1 was measured 24 hrs after 15 μg/g DEN injection to 15-day-old mice by ELISA kit. Control: n≥8; DEN-mice: n≥23. (E) ALT levels in serum were determined 24 hrs after 15 μg/g DEN injection to 15-day-old mice (Control: n=5; DEN-mice: n≥13). The data were analyzed by Student's *t* test. Data are means or means ± SEM.

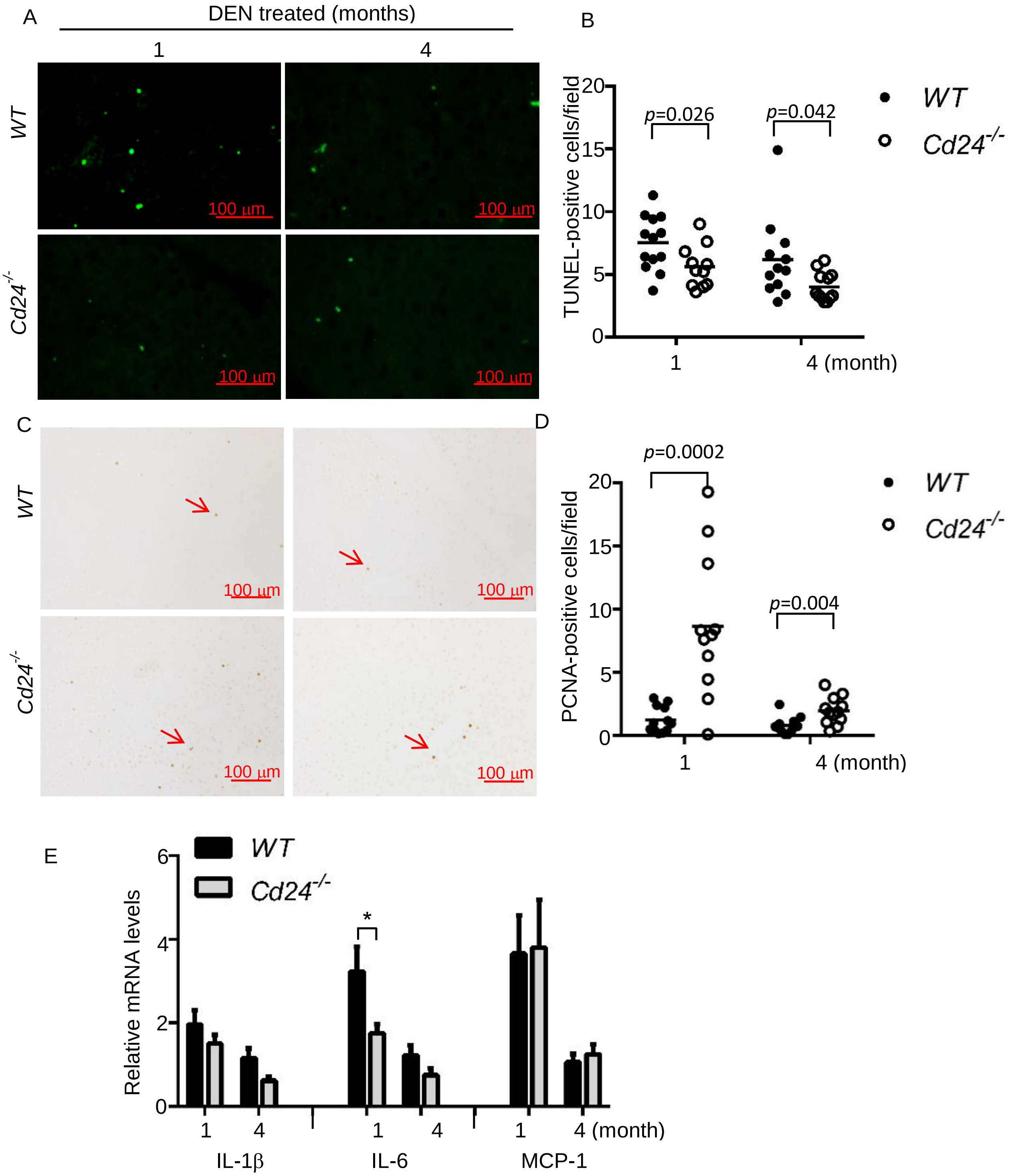

A
DEN treated (months)
1
4
WT
Cd24-/-
100 µm
B
TUNEL-positive cells/field
p=0.026
p=0.042
1
4 (month)
WT
Cd24-/-
C
D
PCNA-positive cells/field
p=0.0002
p=0.004
E
Relative mRNA levels
*
IL-1β
IL-6
MCP-1

Figure S7. Related to Figure 3. Liver tissues were isolated at one or four months after 15 μg/g DEN injection to 15-day-old male mice for histological staining or cytokine measure. (A) Representative liver sections stained by the TUNEL assay. (B) Average numbers of TUNEL-positive cells in a high-power field of a fluroscerence microscope. At least 5 fields from different mice were counted. (C) Representative PCNA staining of liver sections. Arrows denote examples of the PCNA-positive hepatocytes. (D) Average numbers of PCNA-positive cells in a low-power field (× 10) of a microscopy. At least 20 fields from different mice were counted. (E) The levels of some cytokine mRNA were determined by Real-time PCR. ($n \geq 11$). The data were analyzed by Student's *t* test. Data are means or means ± SEM.

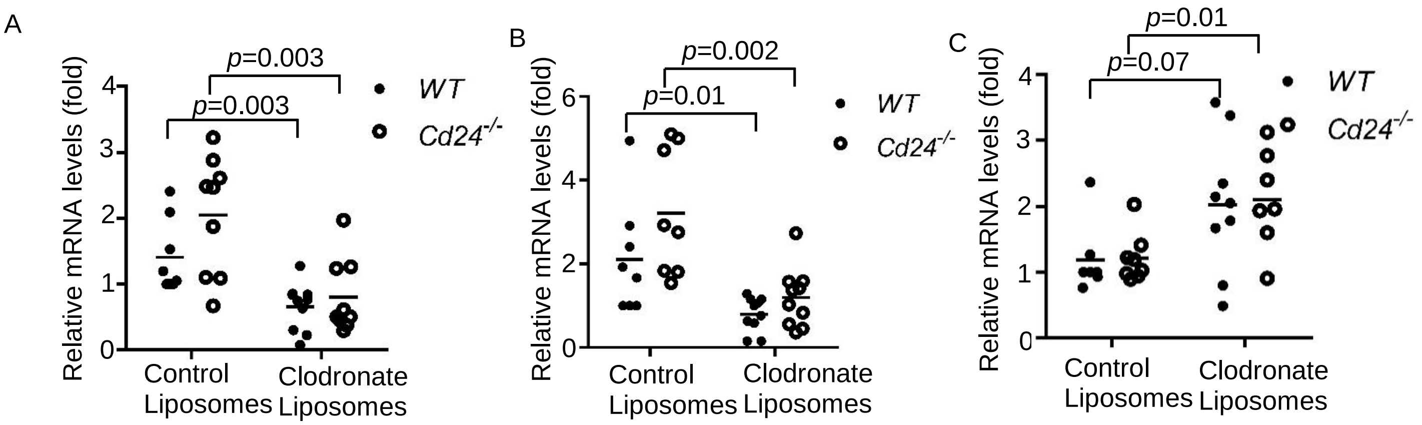

Figure S8. Related to Figure 5. To deplete liver macrophages, 13-day-old mice received i.p. injection of 70 μL of liposomal clodronate or control liposomes at 48 hrs before DEN administration, and then the mice were treated with 15 μg/g DEN. The mRNA levels of cytokine were determined by Real-time PCR. (A) *Il1b*; (B) *Il6*; (C) *Mcp-1*. The data were analyzed by Student's *t* test. Data are means.

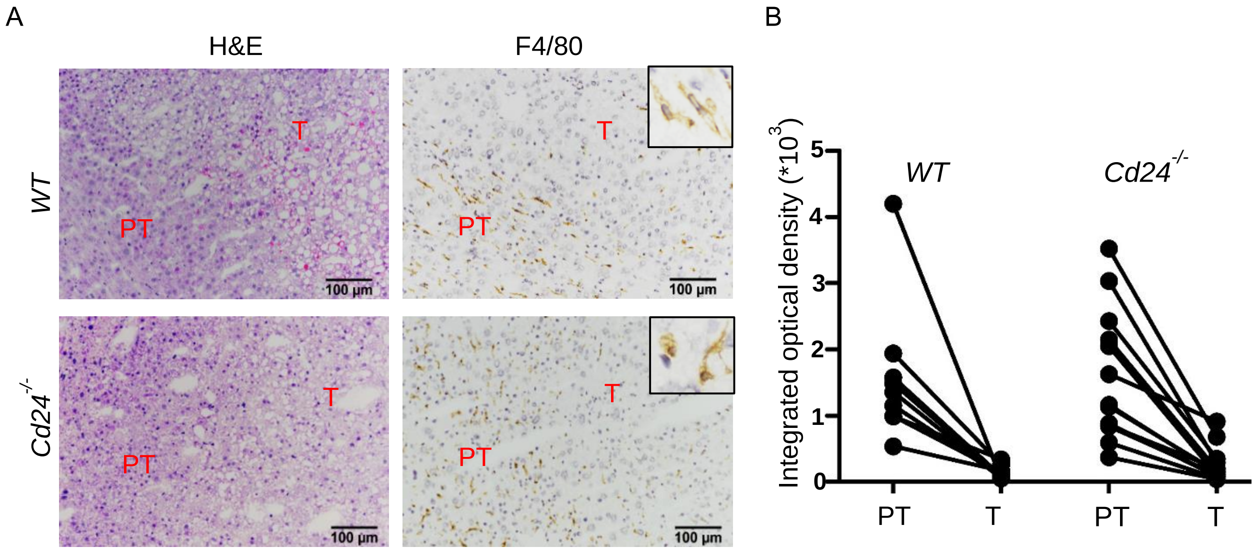

Figure S9. Preferential accumulation of macrophages in peri-tumoral liver regions, related to Figure 5. (A) Representative photographs of H&E and F4/80 staining in peritumoral (PT) and intratumoral (T) liver tissues from DEN-induced HCC mice. Scale bar =100 μm. (B) Integrated optical density (IOD) of F4/80-positive cells quantified by Image-Pro Plus 6.0 software in a lower-power field of a microscopy. At least 5 different fields from each mouse were counted. Data from two independent experiments are pooled.

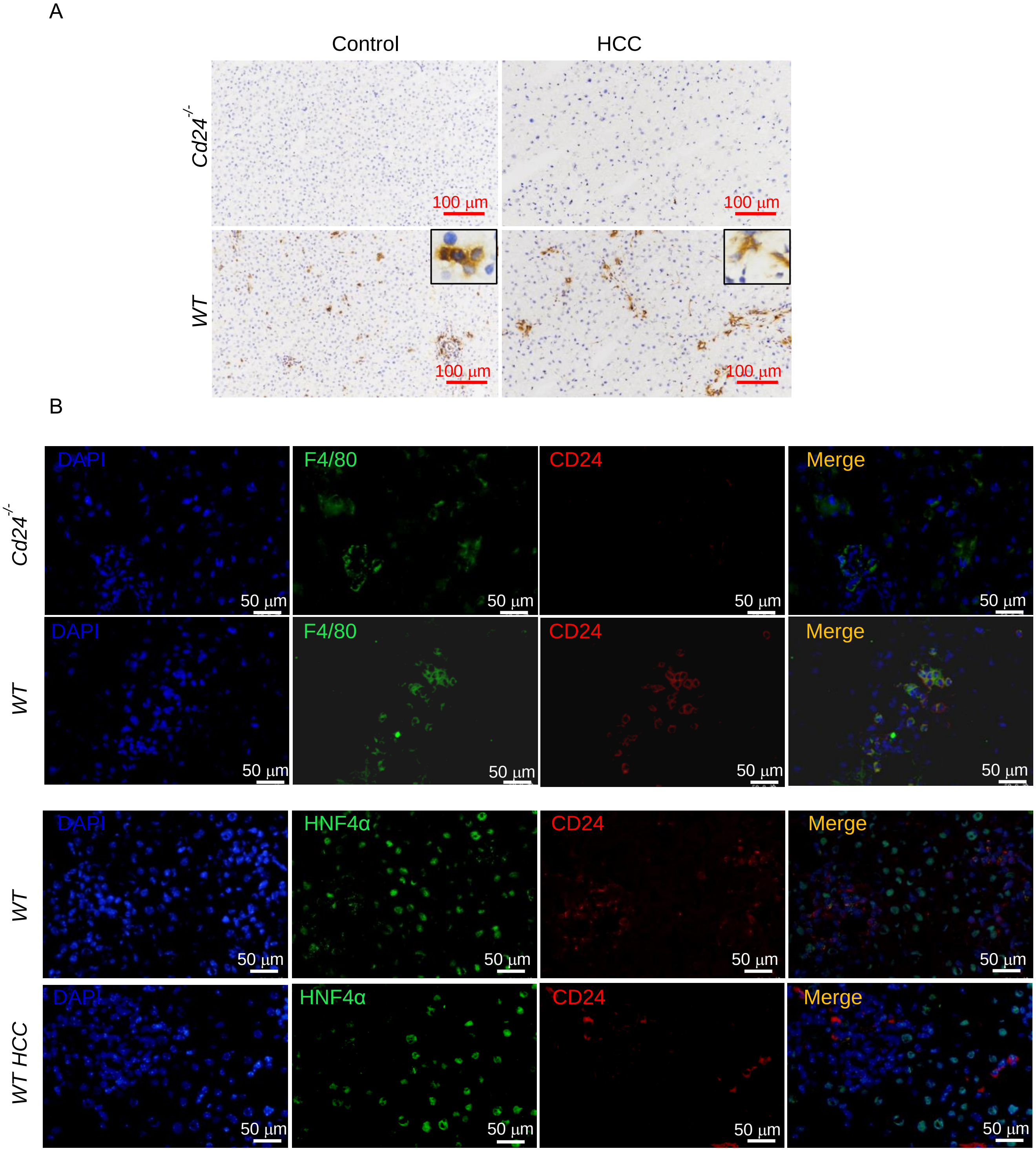
A
Control
HCC
Cd24-/-
WT
100 µm
B
DAPI
F4/80
CD24
Merge
HNF4α
WT HCC
50 µm

C

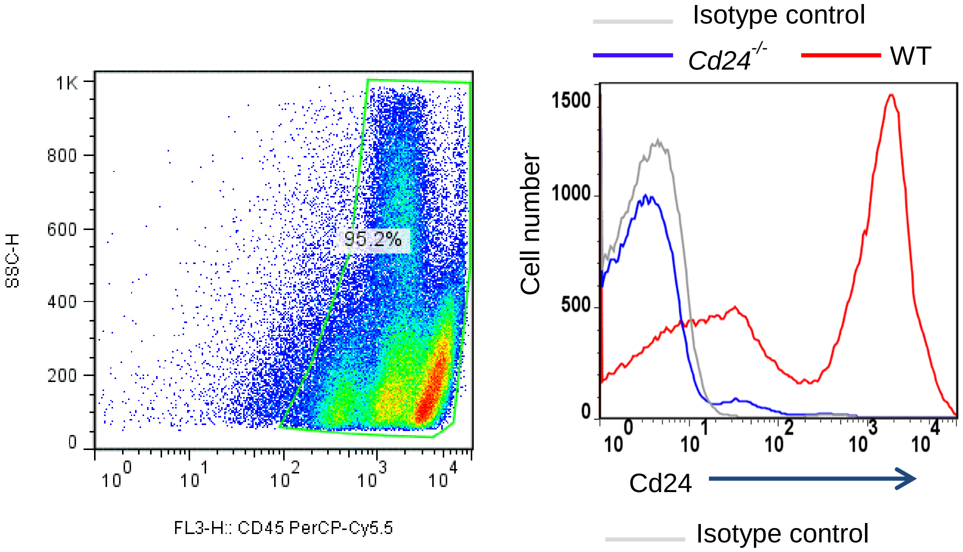

D

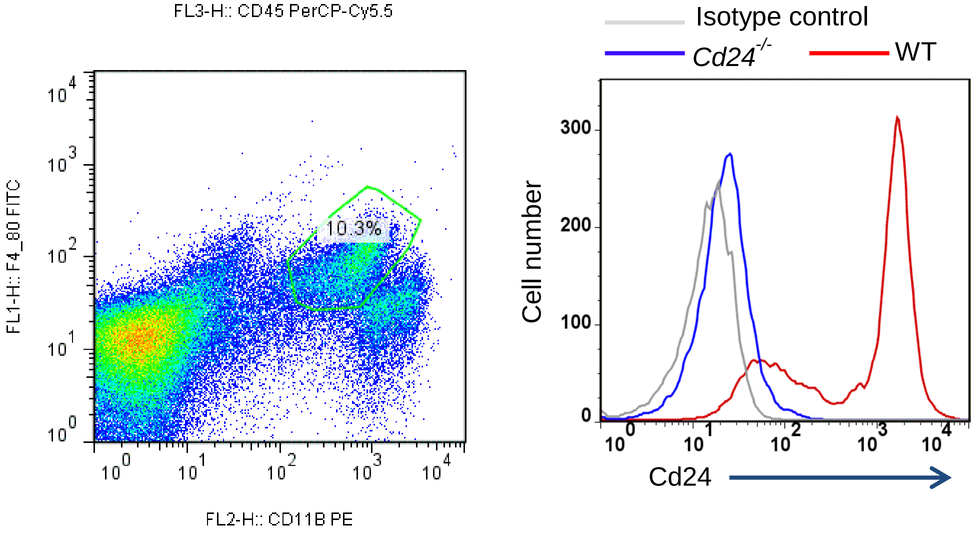

Figure S10. Relates to Figure 6. Cd24 is expressed on intrahepatic mononuclear cells, including macrophages, but not hepatocytes or tumor cells of HCC and naïve mice. (A) Immunohistochemical staining with anti-CD24 antibody. Graphs shown are representative sections of frozen liver samples from naïve (left) and DEN-induced HCC (right) mice. Scale bar =100 μm. (B) Representative immunofluorescent images showing CD24 and F4/80 expression in liver sections of *Cd24*$^{-/-}$ and WT mice (top), CD24 and HNF4α expression in liver sections of naïve and DEN-induced HCC mice (bottom). The cell nuclei

were counterstained with DAPI (blue). Scale bar =50 μm. (C-D) CD24 expression on intrahepatic mononuclear cells by FACS analysis. Mononuclear cells isolated from livers of WT and *Cd24*$^{-/-}$ mice were analyzed by 4-color flow cytometry using antibodies specific for Cd45, Cd24, F4/80 and Cd11b. Representative profiles for gating of leukocytes (C left) and macrophages (D left) and the levels of Cd24 on leukocytes (C right) and macrophages (D right) are presented. Similar data were obtained in two other mice per group.

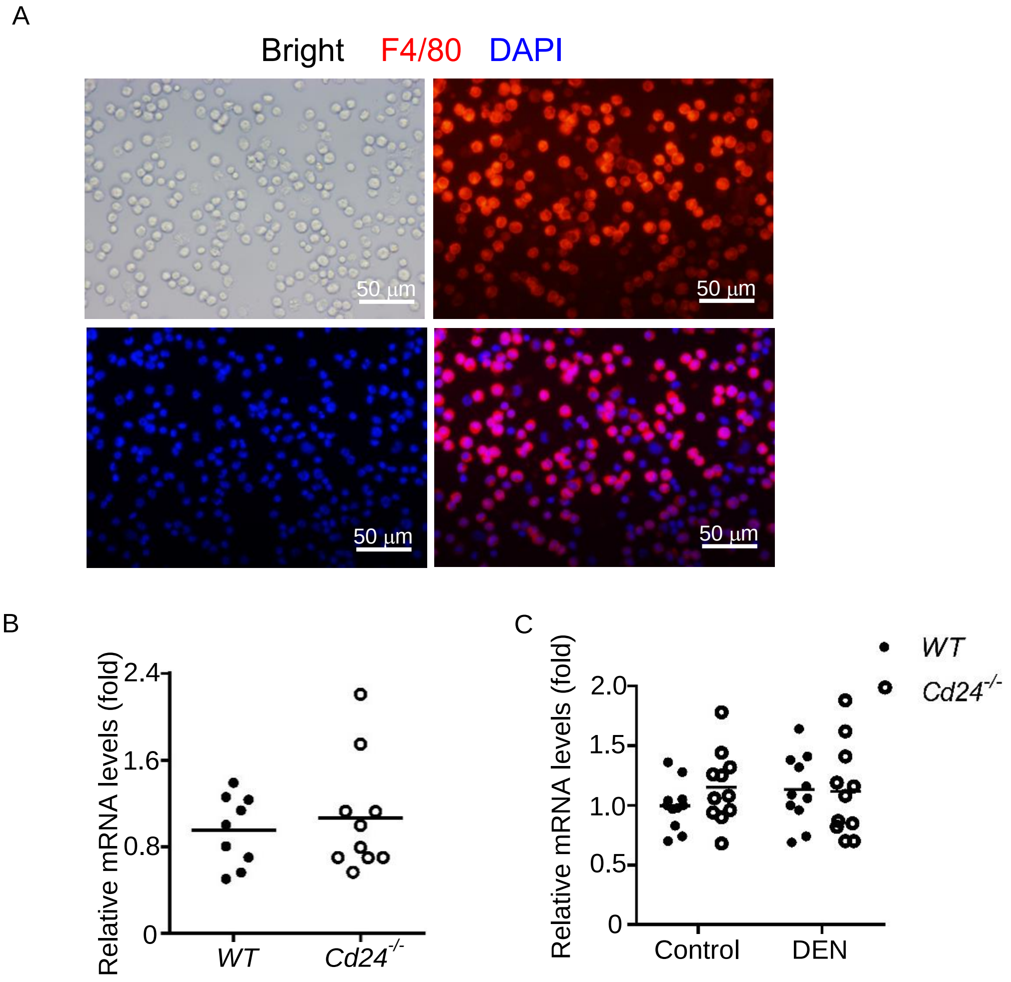

Figure S11. Related to Figure 7. (A). Purity of peritoneal macrophages used for Figure 7. Peritoneal resident cells were incubated for 2 hours to remove the non-adherent cells. The adherent cells were used as source for macrophages which were stained with anti-F4/80 antibodies by immunocytochemical method. (B) The mRNA levels of *puma* after in vivo DEN (15 μg/g) treatment. (C). As in B, except that WT and *Cd24*$^{-/-}$ macrophages were treated with 20 mM DEN in vitro. Data from two independent experiments are pooled. Statistical significance was determined by student t-test.
